# Decreased tubulin-binding cofactor B was involved in the formation disorder of nascent astrocyte processes by regulating microtubule plus-end growth through binding with end-binding proteins 1 and 3 after chronic alcohol exposure

**DOI:** 10.3389/fncel.2022.989945

**Published:** 2022-10-25

**Authors:** Yin Zheng, Mei Yang, Xiaoqiao Chen, Gaoli Zhang, Shanshan Wan, Bingqiu Zhang, Jiechao Huo, Hui Liu

**Affiliations:** ^1^Institute of Neuroscience, Chongqing Medical University, Chongqing, China; ^2^Department of Basic Medicine, Chongqing College of Traditional Chinese Medicine, Chongqing, China; ^3^Institute for Viral Hepatitis, Second Affiliated Hospital of Chongqing Medical University, Chongqing, China; ^4^Department of Blood Transfusion, Sichuan Cancer Hospital and Institute, Chengdu, China; ^5^Fujian Province University Engineering Research Center of Mindong She Medicine, Medical College, Ningde Normal University, Ningde, China

**Keywords:** end binding proteins, ERK1/2 signaling pathway, fetal alcohol syndrome, microtubules, tubulin-binding cofactor B

## Abstract

Fetal alcohol syndrome (FAS) is a neurological disease caused by excessive drinking during pregnancy and characterized by congenital abnormalities in the structure and function of the fetal brain. This study was proposed to provide new insights into the pathogenesis of FAS by revealing the possible mechanisms of alcohol-induced astrocyte injury. First, a chronic alcohol exposure model of astrocytes was established, and the formation disorder was found in astrocyte processes where tubulin-binding cofactor B (TBCB) was decreased or lost, accompanied by disorganized microtubules (MT). Second, to understand the relationship between TBCB reduction and the formation disorder of astrocyte processes, TBCB was silenced or overexpressed. It caused astrocyte processes to retract or lose after silencing, while the processes increased with expending basal part and obtuse tips after overexpressing. It confirmed that TBCB was one of the critical factors for the formation of astrocyte processes through regulating MT plus-end and provided a new view on the pathogenesis of FAS. Third, to explore the mechanism of TBCB regulating MT plus-ends, we first proved end-binding proteins 1 and 3 (EB1/3) were bound at MT plus-ends in astrocytes. Then, through interference experiments, we found that both EB1 and EB3, which formed in heterodimers, were necessary to mediate TBCB binding to MT plus-ends and thus regulated the formation of astrocyte processes. Finally, the regulatory mechanism was studied and the ERK1/2 signaling pathway was found as one of the main pathways regulating the expression of TBCB in astrocytes after alcohol injury.

## Introduction

Fetal alcohol syndrome (FAS), caused by alcohol exposure during pregnancy, is one of the most common causes of nonhereditary lifelong disability worldwide, characterized by widespread impairments in fetal brain structure and function (Carter et al., 2008; Patten et al., 2014). Symptom severity of FAS varies greatly with timing, duration, and dose of alcohol exposure and genetic and metabolic factors (Burd et al., 2003). *In vitro* and *in vivo* models have successfully summarized multiple aspects of the disorder, including morphological and behavioral defects—still, the molecular and genetic mechanisms underlying FAS are poorly understood (Fischer et al., 2021). Astrocytes are the most abundant glial cells in the brain (Han et al., 2020), whose main functions include providing nutrients to the neuron, maintaining the extracellular ion balance, releasing transmitters, regulating synaptic plasticity, maintaining synaptic connection, and so on (Acosta et al., 2017; Palmer and Ousman, 2018; Munger et al., 2019). Astrocytes could not grow normally when cultured in a medium containing alcohol (Renau-Piqueras et al., 1989), and even moderate alcohol could delay their growth and maturation (Renau-Piqueras et al., 1989; Davies and Cox, 1991). They were more sensitive to alcohol than neurons (Lokhorst and Druse, 1993) and were found as one of the main targets of alcoholism during the central nervous system development (Guerri and Renau-Piqueras, 1997). Thus, the morphological and functional changes in astrocytes caused by alcohol toxicity should certainly be related to the pathogenesis of FAS. However, there are few reports about it, and it is worth studying.

Tubulin folding cofactor B (TBCB) participates in the conformational folding of α-tubulin to promote formations of α/β-tubulin heterodimers (Lopez-Fanarraga et al., 2001; Baffet et al., 2012), thus regulating the assembly, growth, and dynamic stability of microtubules (MTs) (Tian et al., 1997; Feierbach et al., 1999; Kortazar et al., 2007; Fleming et al., 2013), which is essential for cell viability. In addition, TBCB has been implicated in neurodegenerative processes (Wang et al., 2005), neurodevelopmental malformations (Tian et al., 2010), human cancer (Vadlamudi et al., 2005), and schizophrenia (Martins-de-Souza et al., 2009). Feltes et al. (2014) also found that the expression of RNA of TBCB was upregulated in adult FAS mice, suggesting that there might be some links between TBCB and the pathogenesis of FAS.

MTs are intrinsically polar filaments with two structurally and functionally distinct ends, the minus- and the plus-end (Desai and Mitchison, 1997; Howard and Hyman, 2003). MT minus-ends connected with the microtubule-organizing center grow slowly, while the plus-ends grow fast and are known as dynamic instability in the dynamic change of polymerization and depolymerization (Mitchison and Kirschner, 1984; Desai and Mitchison, 1997; Nogales and Wang, 2006). TBCB was shown to play a role at MT plus-ends (Lopez-Fanarraga et al., 2007). It was found to co-express with the most abundant MT at the early oocyte edge and (Baffet et al., 2012) to increase neuronal growth cones, which were characterized by active growth of MT plus-end (Lopez-Fanarraga et al., 2007). These data suggested the critical role of TBCB in MT plus-ends. However, TBCB could not directly bind to MT. How does it affect the MT plus-ends?

End-binding proteins (EBS) were key members of MT plus-end tracking proteins (+TIPS) that could directly bind to MT plus-ends and could connect with other proteins, which affected MT plus-ends (Akhmanova and Steinmetz, 2010; Galjart, 2010; Kumar and Wittmann, 2012), including EB1, EB2, and EB3. Among them, EB1 and EB3, but not EB2, promoted continuous microtubule growth (Komarova et al., 2009). TBCB mainly contains three domains, a ubiquitin-like (UBL) domain, a coiled-coil, and a Cytoskeleton-Associated Protein Glycine-rich (CAP-Gly) domain in the C-terminus (Lytle et al., 2004), which can interact with the EEY/F amino acid sequence at the C-terminus of EBs (Carranza et al., 2013). EB1 was preferentially bound to and produced comet-like streaks on the growing MT plus-ends (Sandblad et al., 2006; Dixit et al., 2009) and was found to combine with the mutation of TBCB (TBCBD3) (Carranza et al., 2013). through the EEY/F structure of EB1 and the CAP-Gly domains of TBCB (Honnappa et al., 2006; Steinmetz and Akhmanova, 2008). Similar to EB1, EB3 also has the C-terminal EEY structure (Nehlig et al., 2017) and can theoretically bind with TBCB. Our previous co-immunoprecipitation experiments on Cos7 cells had proved that TBCB could combine with EB3 (data not shown). Therefore, we reasonably speculated that TBCB may affect MT plus-ends through binding to the EB1/EB3 and finally affect the morphology and function of astrocytes. Indeed, it is necessary to conduct a systematic and in-depth study on whether TBCB regulates MT plus-ends through binding with EB1/EB3.

Mitogen-activated protein kinases (MAPK) include extracellular signal-regulated kinase (ERK), c-Jun N-terminal kinase (JNK), and p38 protein kinase (Zhu et al., 2013). Previous studies showed that ethanol can impact the expression of some proteins in neurons (Zamora-Martinez and Edwards, 2014; Qiao et al., 2021), microglia (Gofman et al., 2016), and other cells (Aroor and Shukla, 2004). Alcohol, as an exogenous stimulant, was associated with the MAPK pathway (Zamora-Martinez and Edwards, 2014; Gofman et al., 2016; Qiao et al., 2021), and the ERK1/2 signaling pathway was found sensitive to alcohol (Peng et al., 2009). In addition, long-term alcohol consumption could inhibit the phosphorylation of ERK1/2 but not JNK or p38 in the mesocorticolimbic system (Zhu et al., 2013). In summary, the specific role of the MAPK signaling pathway in TBCB regulation after alcohol exposure needs to be further studied.

To sum up, we speculated that some of the neurological symptoms of FAS should be related to the morphological and functional change of astrocytes caused by TBCB changes, which can regulate MT plus-ends through binding with EB1/EB3 and can be regulated by the MAPK signaling pathway after alcoholic injury. To confirm this, we carried out the following four parts of experiments: first, to study how the astrocytes and TBCB changed and the relationship between their changes after chronic alcohol exposure; second, to confirm whether the astrocytes change is caused by the change in TBCB through silencing or overexpressing TBCB; third, to confirm whether TBCB regulated the MT plus-ends through binding with EB1/EB3 by interrupting their expression and finally led to the morphologic change of astrocytes; and finally, to find and confirm the regulatory pathway of TBCB in astrocytes after chronic alcoholic injury.

## Materials and methods

### Ethics statement

All animals were obtained from the Animal Center of Chongqing Medical University and were approved by the Ethics Committee of Animal Care of Chongqing Medical University. All animal experiments in this study conformed to the standards of the National Institutes of Health Guide for the Care and Use of Laboratory Animals (NIH Publication No. 85-23, revised 1996). The license number for using laboratory animals is SCXK(Chongqing)2018-0003. The experimental animals were killed immediately after purchase, and the primary astrocytes were extracted; all the cells were randomized and blinded. Experimenter blinding was sufficient to control for selection bias. The sample size was evaluated using Power Analysis and Sample Size 2022 (PASS 2022, USA). Furthermore, sample sizes for experiments were sufficient for normality, variance homogeneity, and statistical analyses.

### Primary astrocyte cultures

Mice primary astrocytes were cultured from the cerebral cortices of C57BL/6 mice at postnatal day 0 (Mccarthy and De Vellis, 1980). Briefly, newborn mice were sterilized alive by immersion in 75% ethanol alive, and then the brain tissue was removed and the cerebral membranes were carefully removed. The cerebral cortex was cut into pieces and digested with 0.25% trypsin at 37°C for 10 min, then stopped digestion by a mixture of Dulbecco's modified Eagle's medium with Ham's F-12 medium (LD1223, DMEM/F12, Hyclone) supplemented with 10% fetal bovine serum (900108, FBS, Gemini). After centrifugation, discarded supernate and resuspended the pellet in a mixture of DMEM/F12 supplemented with 10% FBS. Then, the cells' suspension was filtered with a 200-mesh filter (Saimike) and were planted in flasks and incubated at 37°C with 5% CO_2_ and 95% air. The culture medium was replaced every 2 days. After 7 days, the cells were transferred to 6-well plates for western blotting (WB) or Reverse transcription real-time PCR (RT-PCR), and on 12-well plates covered with glass slides for immunofluorescence staining (IF). All experiments were performed on secondary cultures after being grown for 1 day. Cultures of at least 95% astrocytes were used in the following study, as confirmed by immunofluorescent staining (IF) for glial fibrillary acidic protein (GFAP, Supplementary Figure 1A).

### Establishment of chronic alcohol exposure model

Mice secondary-generation astrocytes were randomly divided into a blank control group (Con) and chronic alcohol exposure groups (30, 100 mM). In the chronic alcohol exposure group, secondary astrocytes were cultured in an alcohol-containing medium for 7 days. At 7 days, cells in each group were collected and used for IF (*n* = 6), WB (*n* = 6), and RT-PCR (*n* = 6). The medium with the same alcohol concentration was changed regularly everyday to maintain the alcohol concentration (Ethanol evaporation after 24 h was 10–20%) (Fischer et al., 2021). To analyze the concentration-dependent effect of alcohol, cells were exposed to varying concentrations of alcohol (0, 30,100 mM) (Tomas et al., 2005). The final 30-mM alcohol concentration is similar to the blood alcohol level reported by chronic pregnant drinkers or women weighing about 60 kg when drinking 3–5 glasses of wine within 1 h (Eckardt et al., 1998). Some cells were exposed to 100 mM ethanol for 7 days to analyze the efficacy of high ethanol concentrations (Guasch et al., 2003; Tomas et al., 2003). The alcohol concentrations used are in the range of the blood alcohol levels (BAL) found among alcoholics (Jones and Sternebring, 1992).

### Small-interfering RNA (siRNA) transfection

All small-interfering RNAs (siRNAs) scrambled sequences were synthesized by Chongqing Maobai Technology Co. (Chongqing, China). There was no significant change between the negative siRNA control group and the blank group, so only the negative siRNA control group was displayed as the control group. Astrocytes were transfected with TBCB siRNA or negative oligonucleotides in 6-well or 24-well plates for 6 h using the Lipofectamine™ 3000 transfection kit (L3000015, Invitrogen, USA). Each well of a 6-well plate contained 0.8 × 10^6^ cells, 5 μl siRNA, 3.75 μl Lipofectamine 3000, and 250 μl Opti-MEM (31985062, Gibco, USA). Each well of 24-well plates contained 0.6 × 105 cells per well, 1.25 μl siRNA, 0.75 μl Lipofectamine 3000, and 50 μl Opti-MEM. They were replaced with fresh and DMEM/F12 supplemented with 10% FBS after 6 h. After 48 h following transfection, cells were collected and analyzed by RT-PCR (*n* = 6). After 72 h following transfection, cells were collected and analyzed by WB (*n* = 6) and IF (*n* = 6). All operations were carried out in strict accordance with the commodity instructions. The target siRNA sequences (5 → 3′) used in this study were as follows: TBCB: forward GCAUGGAACUGGAGCUGUATT, reverse UGGUCAAUGACAUGG AUGCTT; EB1: forward GCAGAGGAUUGUAGAUAUUTT, reverse AAUAUCUACAAUCCUCUGCTT; EB3: forward GACGCAAACUAUGAUGGAATT, reverse UUCCAUCAUAGUUUGCGUCTT; and Negative control siRNA: forward UUCUCCGAACGUGUCACGUTT, reverse ACGUGACACGUUCGGAGAATT.

### Lentiviral infection

Mice secondary-generation astrocytes were seeded in 6-well plates with 0.6 × 10^6^ cells. There was no significant change between the no-load lentivirus control group and the blank group, so only the lentivirus no-load control group was displayed as the control group. Astrocytes were randomly assigned to two groups: The control group, cultured in a medium containing 8-μl no-load lentivirus (L2019-358SH GenePharma, Shanghai); and the virus groups cultured in a medium containing 8-μl TBCB overexpress virus (L2019-358SH, EF-1aF/ GFP & Puro, GenePharma, Shanghai) or EB3 overexpress virus (L2019-358SH, EF-1aF/m-Cherry& Puro; GenePharma, Shanghai). After 24 h, a 2-ml DMEM/F12 supplemented with 10% FBS was replaced. After 8 days of infection, cells were collected and analyzed by WB (*n* = 6) and IF (*n* = 6). All operations were carried out in strict accordance with the commodity instructions.

### ERK1/2 signaling pathway interference assay

To confirm whether the ERK1/2 signaling pathway regulated the expression of TBCB after alcohol exposure, the ERK1/2 interference experiment was carried out. Astrocytes were randomly divided into three groups: the solvent control group (Con), cultured in a medium containing 2 μl dissolved in dimethyl sulfoxide (DMSO, Saimike; Zhang et al., 2019); ERK1/2 agonist group (TPA), cultured in a medium containing 200 μM TAP (ERK1/2 agonist, CST; Zhang et al., 2019) dissolved in 2 μl DMSO; ERK1/2 inhibitor group (U0126), cultured in medium containing 10 mM U0126 (MEK1/2 inhibitor, Selleck; Zhang et al., 2019) dissolved in 2 μl DMSO. After 1 h, the drugged medium was removed, and DMEM/F12 supplemented with 10% FBS was added. After 12 h, the three groups of cells were collected separately and analyzed by WB (*n* = 6).

### Cell counting kit-8 multiplication experiment

Cell viability was evaluated using the Cell Counting Kit-8 (CCK-8) assay (AR1199, Boster, China). Mice secondary-generation astrocytes were inoculated on 96-well plates at a concentration of 5 × 10^3^ cells/well (*n* = 6) in 100 μl DMEM-F12 medium containing 10% FBS and cultured at 37°C with 5% CO2 and 95% air. After chronic alcohol exposure, discard the culture medium. A complete medium of 100 μl containing 10 volumes of CCK8 reagent was added to each well and then incubated in the cell incubator for 1 h at 37°C in the dark. The absorbance at 450 nm was measured by the enzyme labeling instrument. Cell viability was calculated using the following equation: cell viability (%) = average OD in the study group/average OD in the control group × 100%.

### Western blotting

The harvested astrocytes were lysed on ice in RIPA cleavage buffer containing 1% PMSF (ST506, Beyotime, Guangzhou, China), and total protein concentration was measured with a BCA protein assay kit (P0012S, Beyotime, China). After dilution in the sample loading buffer, 20 μg of protein was added to each lane. According to the molecular weight of the protein, suitable electrophoresis and wet transfer conditions were selected. The proteins were then separated on a 10% SDS-PAGE gel and transferred to a 0.2-μm polyvinylidene difluoride (PVDF) membrane (IPVH00010, Millipore, USA). The membranes were blocked with a blocking buffer (P0220, Beyotime, China) at room temperature for 30 min. Then, they were probed with the primary antibodies properly diluted overnight at 4°C and then with the HRP-labeled antimouse or antirabbit IgG secondary antibody (ZB-2305 or ZB2301, ZSGBBIO, China) for 1.5 h at room temperature. Then, they were visualized by Western Bright ECL (K-12045-D20, Advansta, USA) and imaged using a Western blotting detection system (Bio-Rad, USA) or X-ray film. There are six repeats, which are three biological repeats multiplied by two technical repeats. Then, the densities of the bands in each image were quantified three times by Quantity-One software. The value of the target protein was normalized to the value of housekeeper protein from the same sample within the same blotting. Then, all the corresponding values from different groups were performed statistically analyzed by GraphPad Prism 6.0 software (GraphPad Software, USA).

The locations of all the proteins detected by the first antibody used in WB were shown in the full-length blotting (Supplementary Figures 1B–N). The location of the first antibody was consistent with that provided by the manufacturer, and the specificity of the first antibody was verified by subsequent silencing or overexpression experiments. The primary antibodies used in WB were listed as follows: Anti-TBCB (1:500, A13248, ABclonal, China), Anti-MAPRE1 (1:1,000, NHA2107, Novogene, China), Anti-MAPRE3 (1:1,000, #AP52003-100, Abcepta, China), Anti-p38 (1:1,000, #8690, Cell Signaling Technology, USA), Anti-pp38 (1:800, #4511, Cell Signaling Technology, USA), Anti-JNK (1:1,000, #9252, Cell Signaling Technology, USA), Anti-p-JNK (1:1,000, #4668, Cell Signaling Technology, USA), Anti-PAK1 (1:1,000, #2602, Cell Signaling Technology, USA), Anti-pPAK1 (1:1,000, #2606, Thermo Fisher Scientific, USA), Anti-β-actin (1:5,000,20536-1-AP, Proteintech, China), Anti-α-T (1: 5,000, GTX628802, GeneTex, USA), Anti-β-T (1:5,000, TA503129, OriGene, USA), Anti-ERK1/2 (1:1,000, #4695, Cell Signaling Technology, USA), Anti-pERK1/2 (1:1,000, #4370, Cell Signaling Technology, USA), and Anti-GAPDH (1:5,000, 60004-1-lg, Proteintech, China).

### Reverse transcription real-time PCR

Total RNA was extracted from harvested astrocytes (*n* = 6) by using RANiso plus (#9108, TaKaRa, China), and the concentration of RNA was measured by spectrophotometer. A total of 1 μg RNA was reverse transcribed to generate cDNA using the PrimeScript™ II 1st Strand cDNA Synthesis Kit (6210A, TaKaRa, China). The expression of messenger of TBCB as a housekeeping gene was assessed by real-time PCR. PCR amplification was performed using a T100 thermal cycler (BIO-RAD) and Premix Taq™ (RR901Q, TaKaRa, China). The PCR reaction mixture consisted of 1 μl of each primer, 25 μl of Premix Taq, and 1 μl of cDNA in a final volume of 50 μl. The PCR conditions were as follows: denaturation at 94°C for 3 min, followed by 34 cycles of denaturation at 94°C for 30 s, annealing at 55°C for 30 s, and extension at 72°C for 30 s. All qPCRs were run on a CFX96 real-time system (Bio-Rad). The 2^−ΔΔCt^ method was used to calculate the fold change of RNA or miRNA level compared to control samples. The primers sequences (5′ → 3′) were listed as follows: TBCB, forward ATGGAGCAGACGACAAGTTCT, reverse CCGTCATCCACAGGATAGGAG, product size (77 bp); EB1, forward AAGCTAGAACACGAGTACATCCA, reverse AGTTTCTTGACCTTGTCTGGC, product size (210 bp); EB3, forward ATGGCCGTCAATGTGTACTCC, reverse GTTTGGCCTGGAACTTCACTT, product size (199 bp); and β-actin (control), forward CAGCCTTCCTTCTTGGGTA, reverse TTTACGGATGTCAACGTCACAC, product size (87 bp). All operations were carried out in strict accordance with the commodity instructions.

### Immunofluorescent staining

The astrocytes were cultured on glass coverslips in a 24-well plate and fixed in −20° precooled acetone and methanol (1:1) for 5 min and then were blocked with 5% bovine serum albumin (BSA) at room temperature for 30 min. The cells were probed with the primary antibodies (anti-TBCB, 1:50, A13248, ABclonal, China; anti-TBCB, 1:250, sc-377139, Santa Cruz, USA; Anti-MAPRE1, 1:1,000, NHA2107, Novogene, China; Anti-MAPRE1, ab53358, Abcam, UK; Anti-MAPRE3, 1:1,000, #AP52003-100, Abcepta, China; anti-α-T, 1: 5,000, GTX628802, GeneTex, USA; GFAP, 1:1,000, P107217, KleanAB, China) properly diluted 4° overnight. Then, the cells were incubated with secondary antibodies (FITC goat antirabbit IgG, 1:200, E031220-01, EARTH, China; Cy3 goat antimouse IgG, 1:200, Abbkine, China) and stained with DAPI (C1005, Beyotime, China). Subsequently, the cells were mounted in Fluorescence Mounting Medium (ab104135, Abcam, UK) and sealed with nail polish. Images were obtained by Inverted fluorescence microscope (Leica DMI8, Germany), and the intensity of fluorescence was analyzed by ImageJ (1.53c) software. The astrocyte processes in high magnification images were also counted by ImageJ (1.53c) software (according to the results, about 50 cells were counted: six 200-fold visual fields were randomly selected in each group, and the cells were counted from the center of the visual field to the periphery). It was mainly used to observe the changes in cell morphology and protein distribution in the study.

### Statistical analysis

Statistical analyses were performed and the corresponding graphs were drawn using GraphPad Prism 6.0 software (GraphPad Software, USA). Data were expressed as means ± standard deviation (SD). The related changes in alcohol intervention groups and control groups were evaluated using a one-way ANOVA. The related changes between other treatment groups and control groups were evaluated using a two-tailed unpaired t-test. The Shapiro–Wilk normality test was performed to analyze the data distribution for each group. Homogeneity of variance was evaluated using Brown–Forsythe and F test for ANOVA and unpaired t-test analyses, respectively. All the data presented in the manuscript passed both tests and were analyzed as normally distributed with equal variances (Supplementary Table 1). Investigators who performed the experiments and the statistical calculations were blinded to the experimental protocol. Values were considered statistically significant when *p* < 0.05 (^*^p < 0.05, ^**^p < 0.01, ^***^p < 0.001, ^****^p < 0.0001).

## Results

### Alcohol inhibited the formation of astrocyte processes and TBCB expression in nascent processes

**Figure 1:**
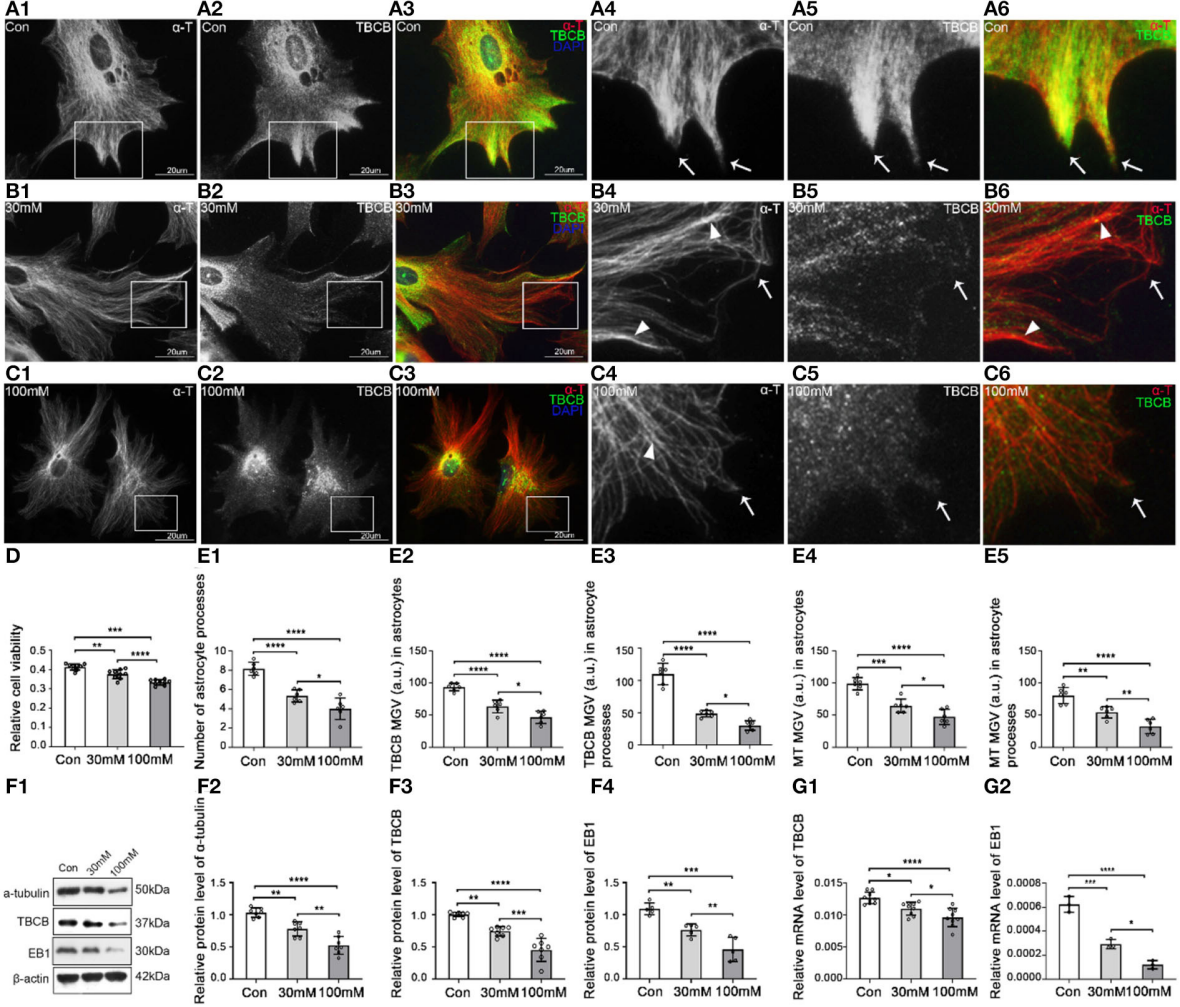
Morphological changes in astrocytes and expression levels of α-tubulin and TBCB after chronic alcohol exposure. **(A–C)** Immunofluorescence showed that α-tubulin (red signal) was co-expressed with TBCB (green signal) in astrocytes both in the control group [Con **(A)**] and the chronic alcohol exposure group [30 mM **(B)**; 100 mM **(C)**]. **(D)** Relative cell viability was detected by Cell Counting Kit-8 after chronic alcohol exposure. **(E)** The number of astrocyte processes and mean gray value (MGV) of TBCB and MT in astrocytes and its processes (MGV = Integrated Density/Area) after chronic alcohol exposure group. **(F)** Western blot and **(G)** RT-PCR analysis of the levels of the α-tubulin protein and TBCB/EB1 protein and its mRNA after chronic alcohol exposure compared with the control group (*n* = 6, *p* < 0.05). **(A–G)** Number of panels. *p < 0.05, **p < 0.01, ***p < 0.001, ****p < 0.0001.

To reveal the changes and the relationship between astrocytes and TBCB after chronic alcohol exposure, low (30 mM) and high (100 mM) ethanol concentrations ethanol were used for establishing chronic alcohol exposure models. Astrocytes in the control group grew well and had good viability (Figure 1D), with flat and plump cell bodies and plenty of processes (Figure 1A). TBCB was mainly not only diffusely distributed in the cytoplasm but also could be arranged in filaments along with MT. It was especially abundant in nascent processes (Figure 1A, arrows), suggesting TBCB played an essential role in the formation of astrocyte processes. Most of the MTs, labeled by α-tubulin, radiated from the organizing center around the nucleus to the edge of the cell cortex and were arranged in dense and regular lines (Figure 1A, arrows). They were especially enhanced in the nascent processes, colocalized with TBCB, hinting at the close relationship between MT and TBCB in this study.

The cell injury was similar in the two alcohol groups, and cell viability decreased (Figure 1D), but the damage degree was concentration-dependent (Figures 1B–D). The astrocytic body collapsed, and the cortical volume and nascent process numbers decreased significantly (Figures 1B,C,E), indicating that alcohol could inhibit astrocyte process formation. The intracellular MT was significantly decreased (Figures 1E,F) with the irregular arrangement, lower density, and more intertwined bundles (Figures 1B,C), especially with the curly plus-ends in the processes (Figures 1B,C, arrowheads), suggesting the irregular growth in MT plus-ends in this study. The expression of TBCB was decreased significantly (Figures 1F,G), particularly in nascent astrocyte processes (Figures 1B,C, arrows, Figure 1E), which were originally in the expression of high-density (Figure 1A, arrows), indicating that alcohol could cause the decrease in the expression of TBCB, especially in astrocytes nascent processes. Moreover, it suggests that the change in the expression of TBCB in this study was closely related to the formation and the growth of nascent astrocyte processes.

### TBCB was involved in the formation of astrocyte processes

**Figure 2:**
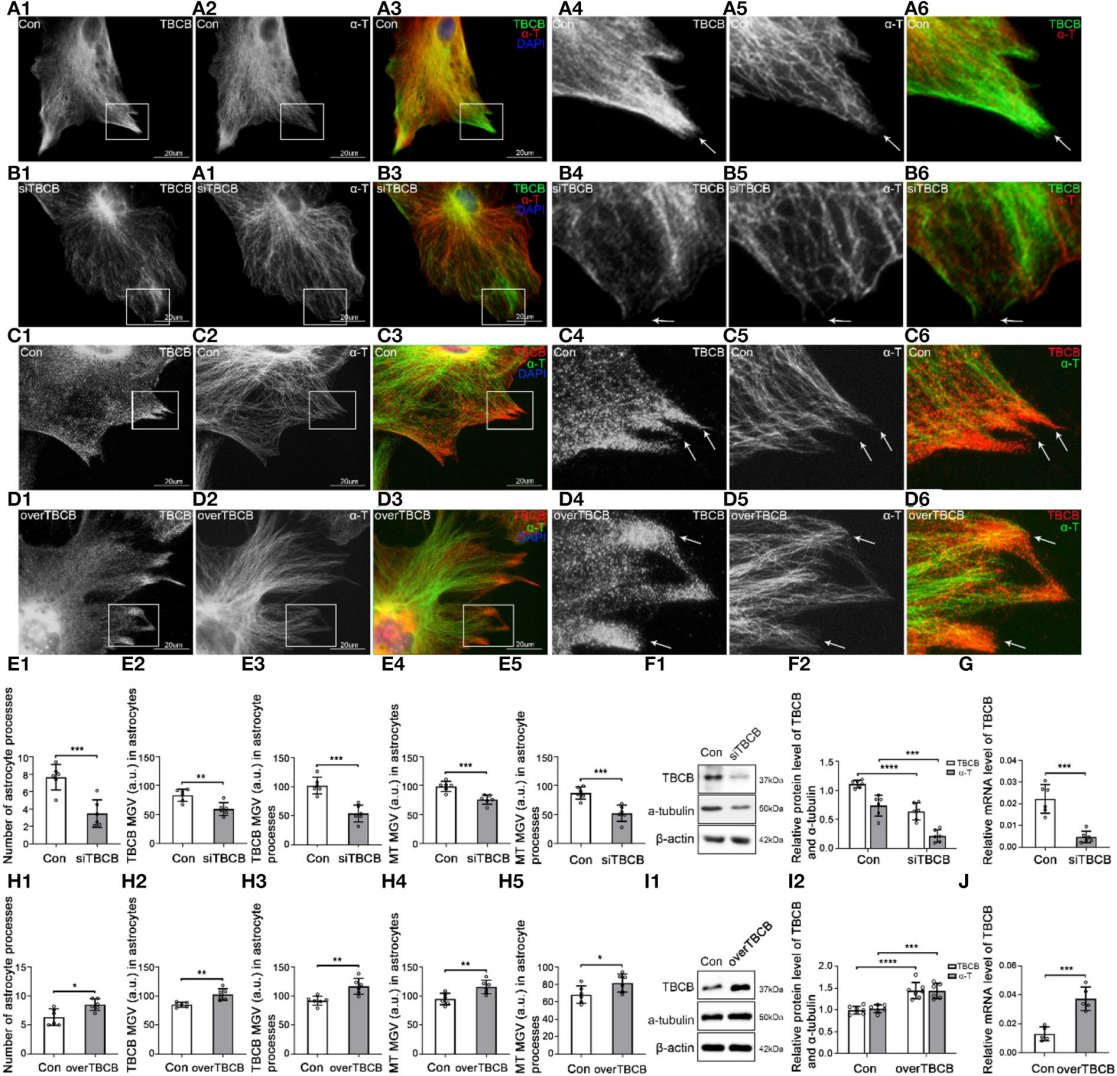
Morphological changes in astrocytes and expression levels of TBCB and α-tubulin after TBCB was silenced or overexpressed. **(A–D)** Immunofluorescence showed that TBCB [**(A,B)** green signal; **(C,D)** red signal] was co-expressed with α-tubulin [**(A,B)** red signal; **(C,D)** green signal] in astrocytes both in the control group [Con **(A)**] and in TBCB silence group **(B)** or TBCB overexpression group **(D)**. **(E,H)** The number of astrocyte processes and mean gray value (MGV) of TBCB and MT in astrocytes and its processes (MGV = Integrated Density/Area) after TBCB was silenced or overexpressed. **(F,I)** Western blot and **(G,J)** RT-PCR analysis of the levels of the α-tubulin protein and TBCB protein and its mRNA after TBCB was silenced or overexpressed compared with the control group (*n* = 6, *p* < 0.05). **(A–J)** Number of panels. ^*^p < 0.05, ^*^* *p* < 0.01, ^*^** *p* < 0.001, ^*^*** *p* < 0.0001.

To confirm the relationship between the decreased expression of TBCB after alcohol exposure and the inhibition of process formation on astrocytes, we used siRNA to silence TBCB. Positive cells transfected by siRNA showed green fluorescence, and the transfection efficiency was more than 90% (Supplementary Figure 2D). WB (Figure 2F) and PCR (Figure 2G) showed that TBCB protein and mRNA content were significantly decreased in the silent group, indicating that the silencing effect was excellent.

After TBCB silence, TBCB still could be observed along with MT (Figure 2B); however, the diffuse TBCB decreased significantly (Figures 2B,E), especially in the nascent astrocyte processes where the original highly expressed TBCB almost disappeared (Figure 2B, arrows, Figure 2E). As well, the intracellular MT content decreased (Figures 2E,F), with the sparse and irregular arrangement, intertwined bundles, and especially the curly plus-ends in nascent processes (Figure 2B, arrows), indicating the irregular growth in MT plus-ends and that the decrease or disappearance of TBCB in nascent processes had severe impacts on the growth of MT plus-ends. With the disappearance of TBCB from nascent processes of astrocytes, most already formed processes were also retracted or disappeared (Figure 2E; Supplementary Figure 2F, arrows). These results showed that TBCB decrease at astrocyte nascent processes did lead to the disorder of astrocyte process formation by regulating the plus-ends of MT.

To understand whether increasing the expression of TBCB had the opposite effect, we also overexpressed TBCB. Lentivirus was used as the vector, and the astrocyte infection rate reached more than 95% (Supplementary Figures 2G,H). WB (Figure 2I) and PCR (Figure 2J) showed that the TBCB protein and mRNA levels were significantly increased in the overexpression group of TBCB, indicating a successful infection. After TBCB was overexpressed, the expression of TBCB increased (Figures 2D,H; Supplementary Figure 2L, arrows), especially in nascent astrocyte processes (Figure 2D, arrows, Figure 2E), accompanied by increased content and dense arrangement of MT in the same place (Figures 2H,I,D, arrows). Meanwhile, astrocyte processes also increased significantly in numbers (Figures 2D,H; Supplementary Figure 2L); however, they lost the original sharp-angle-shape (Figure 2C, arrows), instead with the enlarged basal parts and obtuse tips with the longer extension (Figure 2D; Supplementary Figure 2L, arrows), which was similar to the huge axon-end in Giant Axonal Neuropathy (Wang et al., 2005; Ganay et al., 2011). It was suggested that the accumulation of TBCB could cause the huge processes of astrocytes. All the results of this part indicated that the concentration of TBCB within a certain range was critical for the normal morphology and function of astrocytes. In other words, TBCB was one of the essential factors affecting the formation and growth of astrocyte processes by regulating the growth of MT plus-ends. Combined with the first part experiment, it was proved that the decreased expression of TBCB in astrocyte processes caused by alcohol was one of the main factors leading to the decrease in astrocyte processes by regulating the growth of MT plus-ends. Although how TBCB banded to MT plus-ends and regulated them was still unclear, EB1 and EB3, as the necessary binding proteins, were the potential candidates based on the previous research.

### EB1 and EB3 were bound to MT plus-ends in astrocytes

EB1 and EB3 were reported to bind with MT plus-ends in many kinds of cells (Jaworski et al., 2008), but there were still unclear in astrocytes. To confirm it and pave the way for further experiments, interference experiments were carried out on EB1 and EB3 mediated by siRNA transduction or lentivirus infection. Because that EB1 has been found to bind and regulate MT plus-ends in astrocytes (Bu and Su, 2001; Chiu et al., 2015) and to bind with TBCB directly (Carranza et al., 2013), and also based on the inhibitory results of TBCB and EB1 (Figures 1F,G) on MT plus-ends growth after alcohol injury, we only carried out the silence experiments on EB1 in the following studies. However, little was known about EB3 in the relationship between TBCB and MT plus-ends, so except for silenced experiments, the overexpression experiments of EB3 were also carried out in subsequent studies.

**Figure 3:**
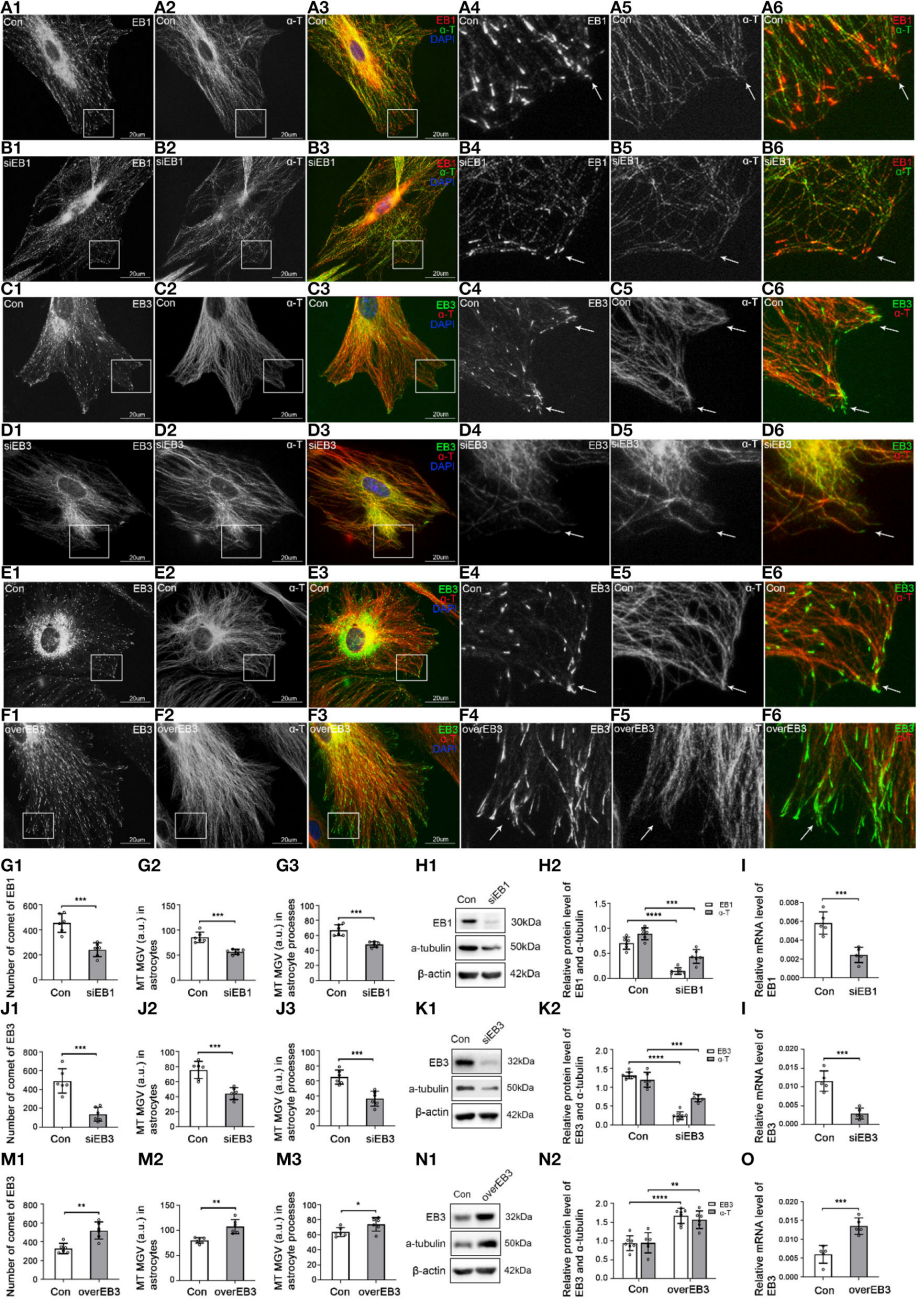
The expression of EB1/EB3 and α-tubulin after EB1/EB3 was silenced or EB3 was overexpressed in astrocytes. **(A–F)** Immunofluorescence showed that α-tubulin [**(A,B)** green signal; **(C–F)** red signal] was co-expressed with EB1 [**(A,B)** red signal] or EB3 [**(C–F)** green signal] in astrocytes both in the control group [Con **(A,C,E)**] and EB1/EB3 silence group **(B,D)** or EB3 overexpression group **(F)**. **(G,J,M)** The number of EB1/EB3 comets and mean gray value (MGV) of MT in astrocytes and its processes (MGV = Integrated Density/Area) after EB1/EB3 was silenced or EB3 was overexpressed. **(H,K,N)** Western blot and **(I,L,O)** RT-PCR analysis of the levels of the α-tubulin protein and EB1/EB3 protein and its mRNA after EB1/EB3 was silenced or EB3 was overexpressed compared with the control group (*n* = 6, *p* < 0.05). **(A–O)** Number of panels. *p < 0.05, **p < 0.01, ***p < 0.001, ****p < 0.0001.

In all the transfection (silence), infection (overexpression), and control groups in our experiments, the transfection and infection efficiencies were more than 90% (Supplementary Figures 2B,C) and 95% (Supplementary Figures 2I,J), respectively. The proteins and mRNA levels of EB1 and EB3 were significantly decreased after gene silencing (Figures 3H,I,K,L) and increased after gene overexpressing (Figures 3N,O), indicating that transfection and infection were successful.

In the control group, IF showed that most MT labeled by α-tubulin originated from perinuclear MT organizing centers and were arranged in a straight, radial, dense, and regular manner (Figures 3A,C,E, arrows), and a few were arranged in a circle along with the cell cortex edge (Figures 3A,C,E). Although EB1 and EB3 could be weakly distributed along with MT, they mainly concentrated on MT plus-ends as typical bright comet-like streaks (Figures 3A,C,E, arrows). Most comets were arranged radially, with the comet-heads located at MT plus-ends and the comet-tails toward MT organizing centers, and a few were arranged on the circle MT plus-ends in the cell cortex. It indicated that EB1 and EB3 were localized at the plus-ends of MT in astrocytes.

After EB1 or EB3 silence, the comets were still located at MT plus-ends, but the numbers decreased significantly (EB1) or even lost (EB3), and changed into thinner, shorter, and smaller comets (Figures 3B,D, arrows, Figures 3G,J). In addition, the content (Figures 3G,I) and numbers (Figures 3B,D,G,J) of MT were decreased, with a sparse, irregular arrangement and curly plus-ends (Figures 3B,D, arrows). The astrocyte cortex was hollowed out and collapsed (Figures 3B,D). These results proved again EB1 localized at MT plus-ends and involved in the regulation of the astrocyte.

After the overexpression of EB3, the comet was still localized at MT plus-ends but significantly denser, longer, and stronger (Figures 3F,M); meanwhile, MT grew exuberantly, mainly in regularly radial straight lines, with obviously increased density and length (Figures 3F,M). The astrocytes were extended and grew dramatically compared with the control group (Figure 3F). Combined with the silence experiments data, EB3 was confirmed as a microtubule plus-end tracking protein in astrocytes and involved in regulating the growth of MT plus-ends.

As we all know, EB1 and EB3 were binding proteins and could not directly act on or regulate MT plus-ends. The regulation function of MT plus-ends they showed was attributed to the proteins they were linked. The above experiments had shown the ability of TBCB to regulate the MT plus-ends. Therefore, TBCB binding to MT plus-ends through EB1/EB3 affects astrocyte processes was our aim for the subsequent study.

### EB1 was necessary to mediate TBCB binding to MT plus-ends

**Figure 4:**
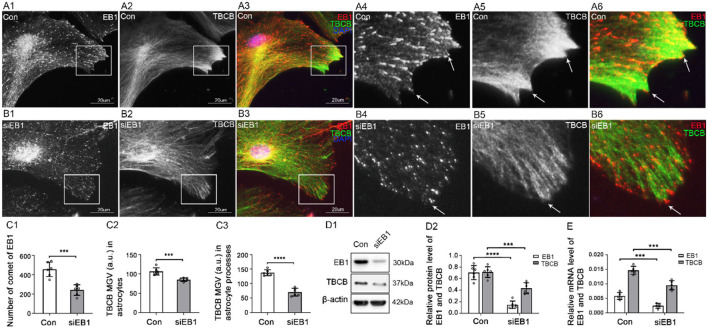
The expression of TBCB after EB1 was silenced in astrocytes. **(A,B)** Immunofluorescence showed that EB1 [**(A,B)** red signal] was co-expressed with TBCB [**(A,B)** green signal] in astrocytes in the negative control group [Con **(A)**] and EB1 silence group **(B)**. **(C)** The number of EB1 comets and mean gray value (MGV) of TBCB in astrocytes and its processes (MGV = Integrated Density/Area) after EB1 was silenced. **(D)** Western blot and **(E)** RT-PCR analysis of the protein and mRNA levels of EB1 and TBCB after EB1 was silenced compared with the control group (*n* = 6, *p* < 0.05). **(A–E)** Number of panels. ***p < 0.001, ****p < 0.0001.

First, to determine whether EB1 was involved in TBCB binding with MT plus-ends in astrocytes, EB1 was silenced with siRNA. EB1 content was decreased (Figures 4C–E), and EB1 comets at MT plus-ends dramatically decreased and shortened into small dots (Figure 4B, arrows, Figure 4C). Furthermore, TBCB content decreased significantly (Figures 4C–E), especially in astrocyte processes, but did not disappear, accompanied by the number decrease in astrocyte processes (Figure 4B, arrows, Figure 4C), in which most of them lost their sharp shape (Figure 4B, arrows). These results indicated that EB1 was necessary to mediate TBCB binding to MT plus-ends, and through this binding, TBCB could regulate the growth of MT plus-ends, thus affecting the formation of the astrocyte process.

### EB3 was necessary to mediate TBCB binding to MT plus-ends

**Figure 5:**
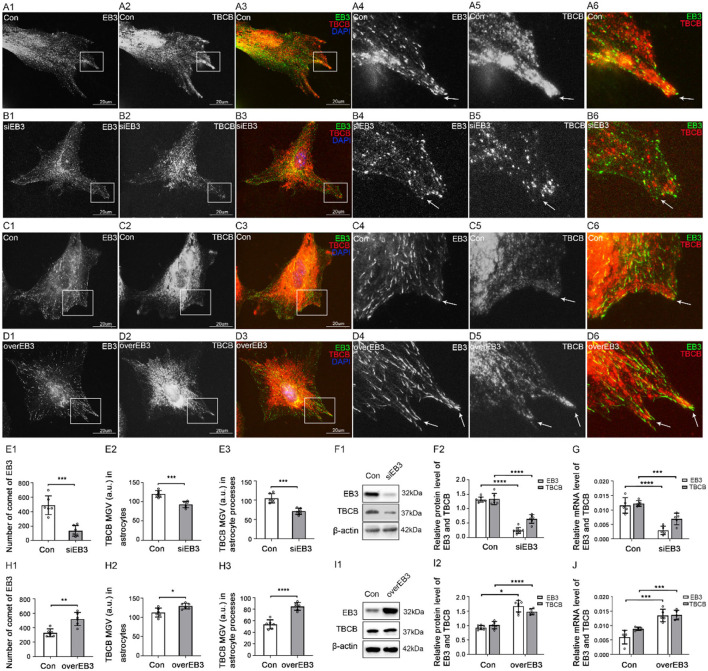
The expression of TBCB after EB3 was silenced or overexpressed in astrocytes. **(A–D)** Immunofluorescence showed that EB3 [**(A–D)** green signal] was co-expressed with TBCB [**(A–D)** red signal] in astrocytes both in the control group [Con **(A,C)**] and EB3 silence group **(B)** or EB3 overexpression group **(D)**. **(E,H)** The number of EB3 comets and mean gray value (MGV) of TBCB in astrocytes and its processes (MGV = Integrated Density/Area) after EB3 was silenced or overexpressed. **(F,I)** Western blot and **(G,J)** RT-PCR analysis of protein and mRNA levels of EB3 and TBCB after EB3 was silenced or overexpressed compared with the control group (*n* = 6, *p* < 0.05). **(A–J)** Number of panels. *p < 0.05, **p < 0.01, ***p < 0.001, ****p < 0.0001.

Second, to determine whether EB3 mediated TBCB binding to MT plus-ends, EB3 silence and overexpression were carried out. After EB3 silence, similar to EB1, the expression of EB3 content was significantly decreased (Figures 5E–G), and the EB3 comets at MT plus-ends were reduced and shortened into small dots (Figure 5B, arrows). In addition, TBCB content decreased significantly, especially in astrocyte processes (Figures 5E,F), and the original high expression of diffused TBCB decreased significantly (Figure 5B, arrows), accompanied by the decrease in astrocyte processes (Figures 5B,E). It proved EB3 was needed for TBCB to perform its normal function on MT plus-ends.

Furthermore, after the overexpression of EB3, EB3 content (Figures 5H–J), and the length, size, and numbers of EB3 comets increased significantly (Figure 5D, arrows, Figure 5H). The expression of TBCB was also increased (Figures 5H–J), and the shape and processes of astrocytes showed no obvious change (Figure 5D). These results further prove that EB3 was necessary for TBCB binding to MT plus-ends and contributing to astrocyte processes.

### EB1 and EB3 changed synchronously

**Figure 6:**
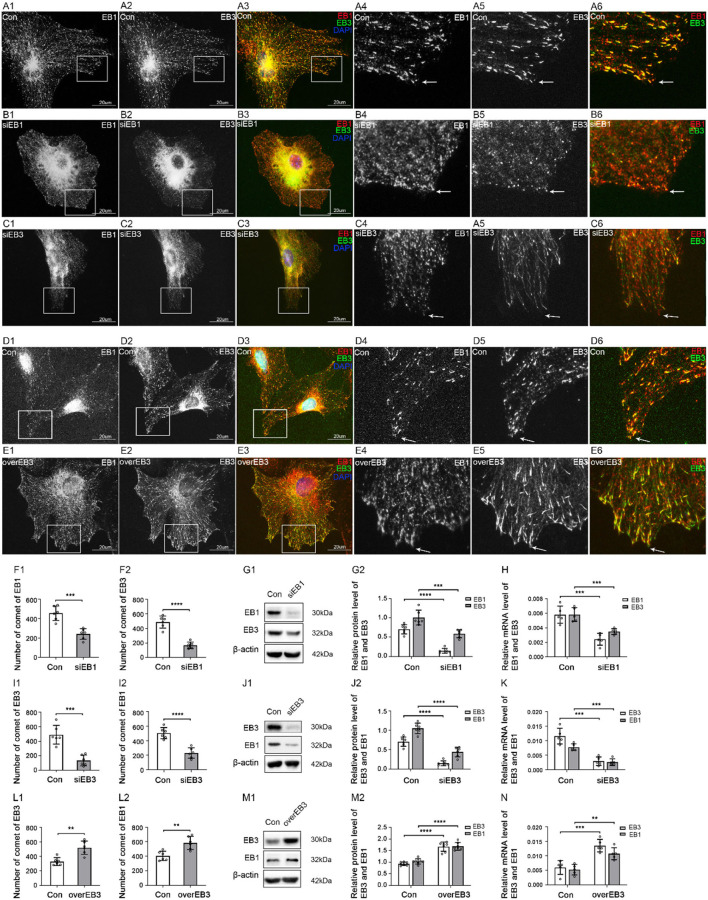
The expression of EB1 and EB3 after EB1/3 was silenced or EB3 was overexpressed in astrocytes. **(A–E)** Immunofluorescence showed that EB1 [**(A–E)** red signal] was co-expressed with EB3 [**(A–E)** green signal] in astrocytes in the control group [Con **(A,D)**], EB1 silence group **(B)**, and EB3 silence group **(C)** or overexpression group **(E)**. **(F,I,L)** The number of comets of EB1 and EB3 in astrocytes after EB1/EB3 was silenced or EB3 was overexpressed. **(G,J,M)** Western blot and **(H,K,N)** RT-PCR analysis of protein and mRNA levels of EB3 and EB1 after EB1/EB3 was silenced or EB3 was overexpressed compared with the control group (*n* = 6, *p* < 0.05). **(A–N)** Number of panels. *p < 0.05, **p < 0.01, ***p < 0.001, ****p < 0.0001.

Thirdly, to explore the possible relationship between the two EB proteins and understand the possible binding model between EB proteins and TBCB, we, respectively, silence and overexpress one EB protein and detect the changes of the other. In addition, EB3 comets co-labeled the tip of EB1 comets at MT plus-ends in the astrocyte control group (Figures 6A,D). After EB1 was silenced, the protein and gene levels of EB3 decreased synchronously with EB1 (Figures 6F–H), the EB3 comets shrank into small dots with the decreased number, and cells' bodies also shrank obviously (Figure 6B, arrows, Figure 6F). These data suggested that the distribution and role of EB3 in MT plus-ends were closely related to the expression of EB1. In addition, after EB3 silence, EB1 protein and gene levels also decreased (Figures 6J,K). Meanwhile, EB1 almost lost the comet shape that changed into small dots and decreased (Figure 6C, arrows, Figure 6I). Furthermore, after EB3 overexpress, the protein and gene levels of EB1 were increased (Figures 6M,N), and EB1 comets also increased significantly and changed longer and stronger (Figure 6E, arrows, Figure 6L). These data suggested that the distribution and role of EB1 in MT plus-ends were also closely related to the expression of EB3. They two changed synchronously and were interdependent, but not complementary, and necessary for each other.

### The change in TBCB expression led to the synchronous change in EB1 and EB3

**Figure 7:**
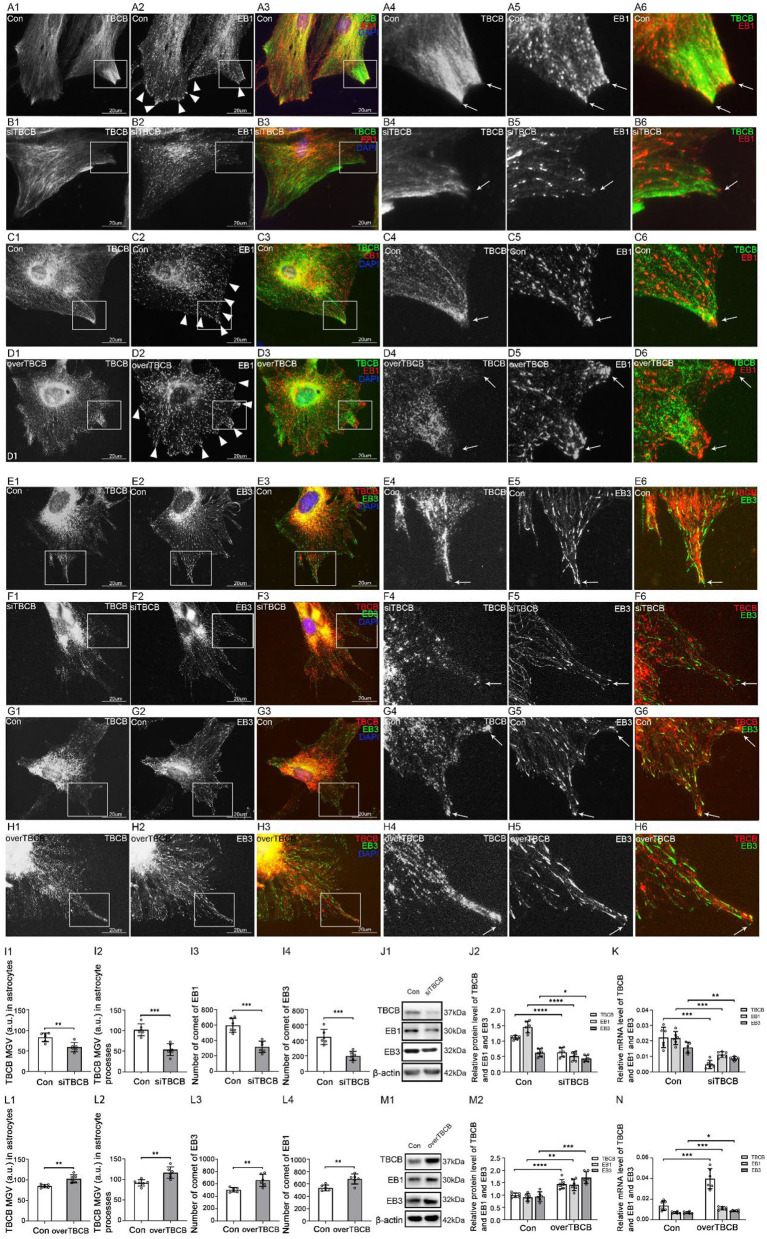
The expression of EB1/EB3 in astrocytes after TBCB was silenced or overexpressed. **(A–H)** Immunofluorescence showed that TBCB [**(A–D)** green signal; **(E–H)** red signal] was co-expressed with EB1 [**(A–D)** red signal] or EB3 [**(E–H)** red signal] in astrocytes both in the control group [Con **(A,C,E,G)**] and TBCB silence group **(B,F)** or TBCB overexpression group **(D,H)**. **(I,L)** The mean gray value (MGV) of TBCB in astrocytes and its processes (MGV = Integrated Density/Area) and the number of EB1/EB3 comets after EB1/EB3 was silenced or EB3 was overexpressed. **(J,M)** Western blot and **(K,N)** RT-PCR analysis of the levels of EB1/EB3 protein and TBCB protein and its mRNA after TBCB was silenced or overexpressed compared with the control group (*n* = 6, *p* < 0.05). **(A–N)** Number of panels. *p < 0.05, **p < 0.01, ***p < 0.001, ****p < 0.0001.

Fourthly, to further explore the relationship between TBCB and EB proteins, TBCB was silenced or overexpressed. After TBCB silence, the expression of TBCB decreased (Figures 7I–K) and almost disappeared in astrocyte processes (Figures 7B,F, arrows, Figure 7I). Conversely, it was abundant in processes after the overexpression of TBCB (Figures 7D,H, arrows, Figure 7L). The expression of EB1 and its mRNA both decreased in the TBCB silence group (Figures 7I–K) and increased in the overexpression group of TBCB (Figures 7L–N), and the comets decreased (Figures 7B,F,I), especially in the edge of astrocytes where the original processes were lost after silence (Figure 7B, arrowheads) or increased significantly in the nascent process after overexpression (Figure 7D, arrows, Figure 7L). It suggested that the change in EB1 was a passivity response to the disorder of the process formation due to the irregular growth of MT plus-ends caused by TBCB changes.

The expression of EB3 and its mRNA both decreased in the TBCB silence group (Figures 7I–K) and increased in the overexpression group of TBCB (Figures 7L–N). However, its comets were almost lost and changed into small dots after TBCB silencing (Figure 7F, arrows, Figure 7I) or increased obviously and changed bigger and longer after TBCB overexpressing (Figure 7H, arrows, Figure 7L). It suggested that EB3 was not only involved in binding with TBCB on MT plus-ends but also relied, at least partly, on TBCB to execute its normal function.

The above experiments confirmed that alcohol could cause TBCB decrease, impede the growth of MT plus-ends through binding with EB1 and EB3, and cause the deficient arrangement of astrocyte processes, finally leading to neurological symptoms. However, the signaling pathway that regulates the expression of TBCB after alcohol exposure is still unclear, and we did the following experiments to explore it.

### ERK1/2 signaling pathway regulated the expression of TBCB

**Figure 8:**
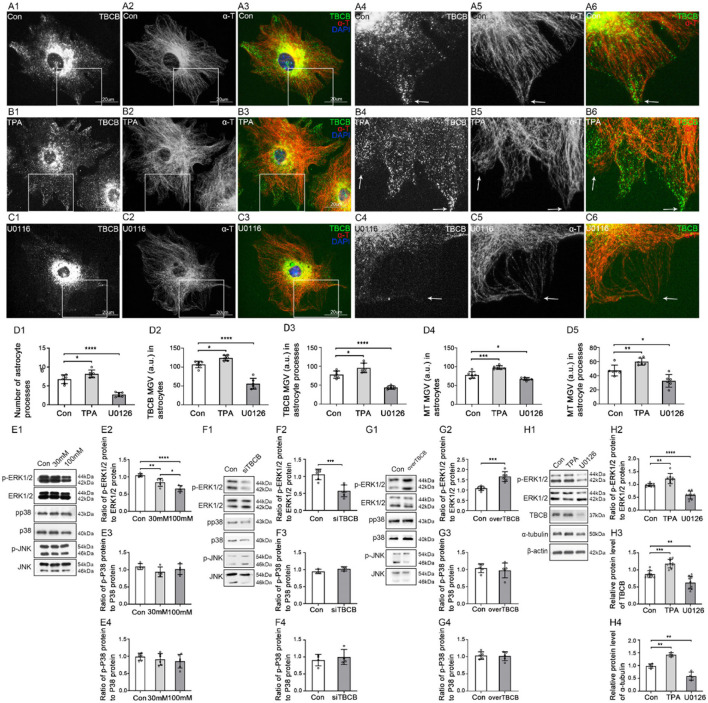
Changes in protein levels in MAPK signaling pathway after chronic alcohol exposure or TBCB interference, and distribution and protein levels of TBCB and α-tubulin in astrocytes after interfering with ERK1/2 signaling pathway. **(A–C)** Immunofluorescence showed that TBCB (green signal) was co-expressed with α-tubulin (red signal) in astrocytes both in the control group [Con **(A)**] and ERK1/2 agonist group [TPA **(B)**] or ERK1/2 inhibitor group [U0126, **(C)**]. **(D)** The number of astrocyte processes and mean gray value (MGV) of TBCB and MT in astrocytes and its processes (MGV = Integrated Density/Area) after interfering with the ERK1/2 signaling pathway. **(E–G)** Western blot analysis of phosphorylation levels and relative quantitative analysis of MAPK (ERK1/2, p38, JNK) signaling pathways in astrocytes under various interfering factors (*n* = 6, *p* < 0.05). **(H)** Western blot analysis of the ratio of p-ERK1/2 to ERK1/2, and protein levels of TBCB and α-tubulin in astrocytes after TPA pretreatment or after U0126 pretreatment (*n* = 6, *p* < 0.05). **(A–H)** Number of panels. *p < 0.05, **p < 0.01, ***p < 0.001, ****p < 0.0001.

To determine whether the MAPK signaling pathway regulates the expression of TBCB, we detected MAPK phosphorylation levels after chronic alcohol exposure to astrocytes (Figures 8E–G). Compared with the control group, the p-ERK1/2 protein level decreased after chronic alcohol exposure (Figure 8E, *p* < 0.05), which was consistent with the change of TBCB, while p-P38 and p-JNK showed no significant change (Figure 8E, *p* > 0.05). It suggested that the MAPK-ERK1/2 signaling pathway might interfere with the expression of TBCB after chronic alcohol exposure. Therefore, to further confirm the relationship between the ERK1/2 pathway and TBCB, the phosphorylation levels of MAPK were detected after silencing or overexpressing TBCB. Similar results were gathered: p-ERK1/2 was changed synchronously with that of the TBCB (Figures 8F,G, *p* < 0.05) but not of the p-P38 and p-JNK (Figures 8F,G, *p* > 0.05). It indicated that the ERK1/2 signaling pathway was involved in the regulation of the expression of TBCB after chronic alcohol exposure.

Moreover, to confirm that the ERK1/2 signaling pathway was one of the main signaling pathways regulating the expression of TBCB, astrocytes were treated, respectively, with ERK1/2 agonist (TPA) and MEK1/2 inhibitor (U0126) (Figures 8A–C,H). The expression of TBCB and α-tubulin upregulated (Figure 8H, *p* < 0.05) with special abundant TBCB and densely messy MT in newly formed processes (Figure 8B, arrows, Figure 8D) after activating the ERK1/2 pathway. In contrast, they were downregulated (Figure 8H, *p* < 0.05) with scarce TBCB and irregular MT in decreased astrocyte processes (Figure 8C, arrows, Figure 8D) after inhibiting the ERK1/2 pathway. These results proved that activation or inhibition of the ERK1/2 signaling pathway could change the expression of TBCB and then affect the formation of astrocyte processes. To sum up, the MAPK-ERK1/2 signaling pathway was one of the main signaling pathways to regulate the expression of TBCB in astrocytes after alcohol exposure.

## Discussion

In this study, we confirmed that the decreased TBCB was one of the critical factors for the formation and growth disorder of astrocyte processes after chronic alcohol exposure; TBCB, which could be regulated by ERR signaling pathway, regulated the growth of MT plus-ends through binding with EB1 and EB3, and thus regulated the formation and growth of astrocyte processes. Besides these, our study also showed the following interesting findings.

### TBCB content in a certain range was critical for the normal morphology of astrocyte processes

Tubulin cofactor B was initially discovered as a protein required for proper tubulin folding and heterodimer formation (Lopez-Fanarraga et al., 2007; Baffet et al., 2012), transitory tubulin storage (Tian et al., 1997), tubulin degradation processes (Keller and Lauring, 2005; Kortazar et al., 2007), and the synthesis, growth, and metabolism of MT (Carranza et al., 2013; Tian and Cowan, 2013; Nithianantham et al., 2015; Al-Bassam, 2017). Previous experiments found two forms of the expression of TBCB: mainly distributed diffusely in the cytoplasm (Feierbach et al., 1999; Radcliffe et al., 1999; Kortazar et al., 2007; Lopez-Fanarraga et al., 2007) and partly overlaps with MT (Vadlamudi et al., 2005; Baffet et al., 2012), which were similar to our findings in astrocytes. TBCB was found localized on newly polymerized microtubules (Vadlamudi et al., 2005; Carranza et al., 2013) and might act on MT plus-ends in oocytes (Baffet et al., 2012), microglia (Fanarraga et al., 2009a,b), and neurons (Mitchison and Kirschner, 1984), thus affecting cell polarity, dynamic changes, and neurite growth. Our study also supplied similar data about astrocytes.

However, for the role of TBCB to MT plus-ends, maybe due to species or cells diversity and different degrees of TBCB interference or difference in technical approaches, there were inconsistent results: decreased or lost TBCB resulted in the decrease in α-tubulin (Radcliffe et al., 1999; Radcliffe and Toda, 2000; Baffet et al., 2012) vs. enhancing MT density (Lopez-Fanarraga et al., 2007; Fanarraga et al., 2009b); the overexpression of TBCB led to MT loss (Radcliffe et al., 1999; Wang et al., 2005; Kortazar et al., 2007; Fanarraga et al., 2009b; Ganay et al., 2011; Baffet et al., 2012) vs. obvious enhancement on the MT plus-ends growth in ours study; TBCB was not a destabilizing agent of MTs (Cleveland et al., 2009; Ganay et al., 2011). In this study, normally, TBCB was highly expressed in the new processes of astrocytes and co-expressed with MT plus-ends. In our study, silencing or overexpressing TBCB induced the synchronous change in MT plus-ends (Figure 3), which both led to the disorder of processes formation: the former impeded the formation or growth of astrocyte processes and the latter enhanced the formation or growth of astrocyte processes in the abnormal shapes with expanding basal parts and obtuse tips (Figure 2D; Supplementary Figure 2L, arrows), like the enlargement of the neuron axon in Giant Axonal Neuropathy (Ganay et al., 2011). Our data indicated that the balance of TBCB within a certain range was critical for the normal morphology and function of astrocytes; too low a concentration of TBCB was not conducive to the formation and growth of astrocyte processes, and too high a concentration would lead to expanding at the process base, which may be achieved by participating in the assembly and dissociation of MT plus-ends.

### TBCB might affect the stability of MT minus-ends and MT walls

In most mammalian cells, microtubules grow from the microtubule-organizing center (MTOC) near the nucleus (usually the centrosome), where their minus-ends may be stably anchored (Schuyler and Pellman, 2001; Dammermann et al., 2003; Louie et al., 2004; Galjart, 2010). TBCB was found to localize at the centrosome of the base of the primary cilium (Bloodgood, 2009; Carranza et al., 2013) and the centrosome of Vero cells (Vadlamudi et al., 2005). When TBCB of astrocytes decreased due to alcohol interference and siRNA silencing, the perinuclear TBCB did not change significantly (Figures 1A–C, 2A–D), and there was still TBCB distributed along with the MT (Figures 1A–C, 2A–D), indicating that perinuclear TBCB and TBCB arranged along with MT might have an additional function. MT shaft was composed of GDP-tubulin and was intrinsically unstable (Akhmanova and Steinmetz, 2015). These results indicated that TBCB might be related to the stability of the MT wall and minus-ends, while the specific effect needs to be further discussed.

### EB1 and EB3 might bind to MT plus-ends as a heterodimer in astrocytes

Our study showed that EB1 and EB3 were always changed synchronously in astrocytes. Silencing one of them, led the both to decrease with the typical comets losing or changing into small dots (Figures 6B,C, arrows); overexpressing EB3 led them both to upregulate with bigger, longer, and more comets (Figure 6E). Although EBs could compete with each other (Komarova et al., 2009) in astrocytes, there was undoubtedly not in this case. In addition, although our results showed that they were both required for TBCB binding to MT plus-ends, they also did not show the character of compensating each other for binding with TBCB when one of them was decreased or lost. The findings that EB1 and EB3 could form heterodimers, which have a higher affinity for the growing of MT plus-ends than single monomers in cells (Komarova et al., 2009), could explain our results. Therefore, it can be concluded, based on our and the previous data, that EB1 and EB3 may bind to MT plus-ends as a heterodimer in astrocytes. This speculation also was confirmed by the results that silencing or overexpressing any one of these EBs always led to the synchronous change of TBCB (Figures 4, 5). It also hinted that TBCB connected with MT plus-ends might be through the heterodimer of EB1 and EB3. However, further experiments are still needed to verify it.

### Accumulated TBCB, not EB3, was involved in the formation of the huge nascent processes in astrocyte

TBCB and EB3 were both increased obviously after the overexpression of TBCB or EB3. However, the shape of nascent astrocyte processes was different: maintaining the original shape after the overexpression of EB3 (Figures 5D, 6E) vs. changing into the expanded basal parts with the obtuse tips after the overexpression of TBCB (Figures 7D,H). After EB3 overexpressing, EB1 was increased obviously (Figure 6D) for forming the heterodimer with EB3, and all binding partners connected to MT plus-ends through EBs, including TBCB, were increased responsively and passively. These proteins increased proportionally, finally inducing more processes with the normal shape.

However, overexpressing TBCB led to the abnormal nascent process—“huge process,” which has the expanded basal part with an obtuse tip (Figure 2D; Supplementary Figure 2L, arrows). Although EB1 and EB3 were increased responsively (Figure 7), other partners required to build the processes could not be regulated proportionally or could not compete with accumulated TBCB for the binding site, led to abnormal growth of MT plus-ends and finally caused the malformed nascent processes in astrocyte. These results also proved the special function of TBCB in regulating the formation and growth of nascent astrocyte processes.

### TBCB was involved in the formation of EB3 comet

After TBCB silencing or overexpressing, the content and the comets number of EB1 and EB3 were changed synchronously (Figure 7), possibly because of the response to the change of TBCB. But interestingly, the shape of their comets was changed inconsistently. The EB1 comets still maintained their original shape (Figures 7B,D); however, most EB3 comets changed into smaller dots after TBCB silence (Figure 7F) or became more prominent and stronger after TBCB overexpressing (Figure 7H). These proved that TBCB might not involve in the comet structure formation of EB1 but might participate in EB3s. In other words, the formation of EB3 comets, a symbol of active function in normal (Mourino-Perez et al., 2013), might depend, at least partly, on the expression of TBCB. However, further and systemic research is still needed to reveal it.

## Conclusion

Some of the neurological symptoms of FAS might be related to the formation disorder of astrocyte processes, and the altered organization of astrocytes reported in the FAS could be explained by the decrease in TBCB which could regulate the growth of MT plus-ends through binding with EB1/EB3 and by MAPK-ERK1/2 signaling pathway. These observations have relevance for understanding the mechanism underlying the astrocyte alterations that occurred in the pathogenesis of fetal alcohol syndrome.

## Data availability statement

The original contributions presented in the study are included in the article/Supplementary material, further inquiries can be directed to the corresponding author.

## Ethics statement

The animal study was reviewed and approved by the Ethics Committee of Animal Care of the Chongqing Medical University.

## Author contributions

HL and MY designed experiments. YZ and G-LZ performed experiments. YZ, S-AW, X-QC, B-QZ, and J-CH contributed to the data analysis. YZ wrote the main manuscript text. HL modified the manuscript. All authors reviewed the manuscript. All authors contributed to the article and approved the submitted version.

## Funding

This work was supported by the National Natural Science Foundation of China (81971230, 81500978, 81671312, and 81000566) and the Natural Science Foundation Project of Chong Qing (cstc2016jcyjA0229, cstc2017jcyjAX0414, cstc2015jcyja10018, and cstc2011jjA10093) and the Foundation of Chongqing Municipal Education Commission (KJ1600213).

## Conflict of interest

The authors declare that the research was conducted in the absence of any commercial or financial relationships that could be construed as a potential conflict of interest.

## Publisher's note

All claims expressed in this article are solely those of the authors and do not necessarily represent those of their affiliated organizations, or those of the publisher, the editors and the reviewers. Any product that may be evaluated in this article, or claim that may be made by its manufacturer, is not guaranteed or endorsed by the publisher.
