# Supplementary material for: Decreased tubulin-binding cofactor B was involved in the formation disorder of nascent astrocyte processes by regulating microtubule plus-end growth through binding with end-binding proteins 1 and 3 after chronic alcohol exposure
Source: Front Cell Neurosci. 2022 Oct 25;16:989945. doi: 10.3389/fncel.2022.989945 (PMC9641617; doi:10.3389/fncel.2022.989945)
Supplement: Supplementary file 1 [file Data_Sheet_1.docx]

Supplementary Material

## Supplementary Figures


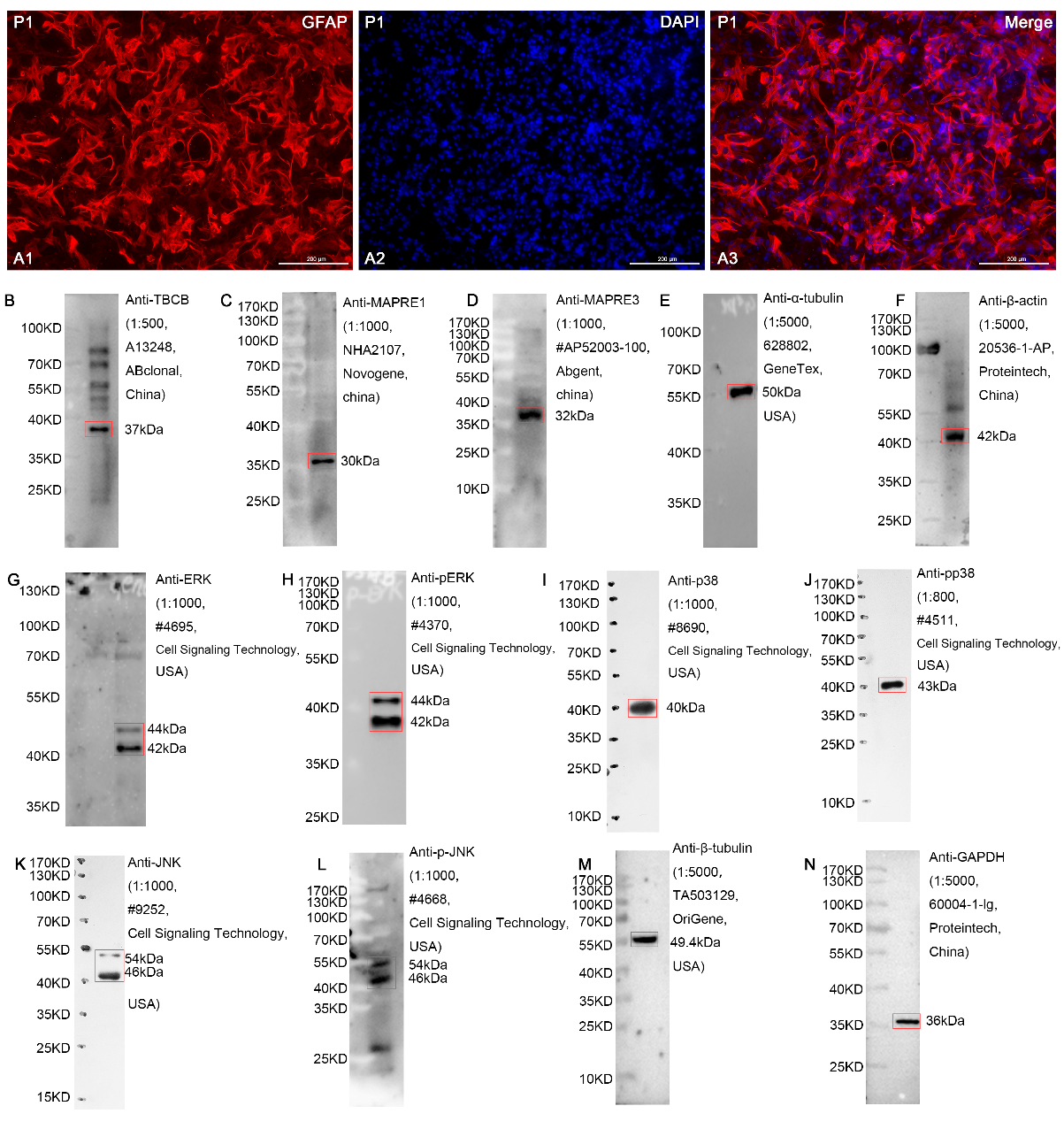


**Supplementary Figure 1.** Purity identification of the mice second-generation astrocytes, and localizations of all the proteins in this study shown in full-length Western Blot image. **(A1-3)** The purity of mice second-generation astrocytes was more than 90% by Immunofluorescent staining for glial fibrillary acidic protein (GFAP), **(B)** Anti-TBCB (1:500, A13248, ABclonal, China), **(C)** Anti-MAPRE1(1:1000, NHA2107, Novogene, china), **(D)** Anti-MAPRE3 (1:1000, #AP52003-100, Abcepta, china), **(E)** Anti-α-T(1：5000， GTX628802, GeneTex, USA), **(F)** Anti-β-actin (1:5000,20536-1-AP, Proteintech, China), **(G)** Anti-ERK1/2 (1:1000, #4695, Cell Signaling Technology, USA), **(H)** Anti-pERK1/2 (1:1000, #4370, Cell Signaling Technology, USA), **(I)** Anti-p38 (1:1000, #8690, Cell Signaling Technology, USA), **(J)** Anti-pp38 (1:800, #4511, Cell Signaling Technology, USA), **(K)** Anti-JNK (1:1000, #9252, Cell Signaling Technology, USA), **(L)** Anti-p-JNK (1:1000, #4668, Cell Signaling Technology, USA), **(M)** Anti-β-T (1:5000，TA503129, OriGene, USA), **(N)** Anti-GAPDH (1:5000, 60004-1-lg, Proteintech, China).


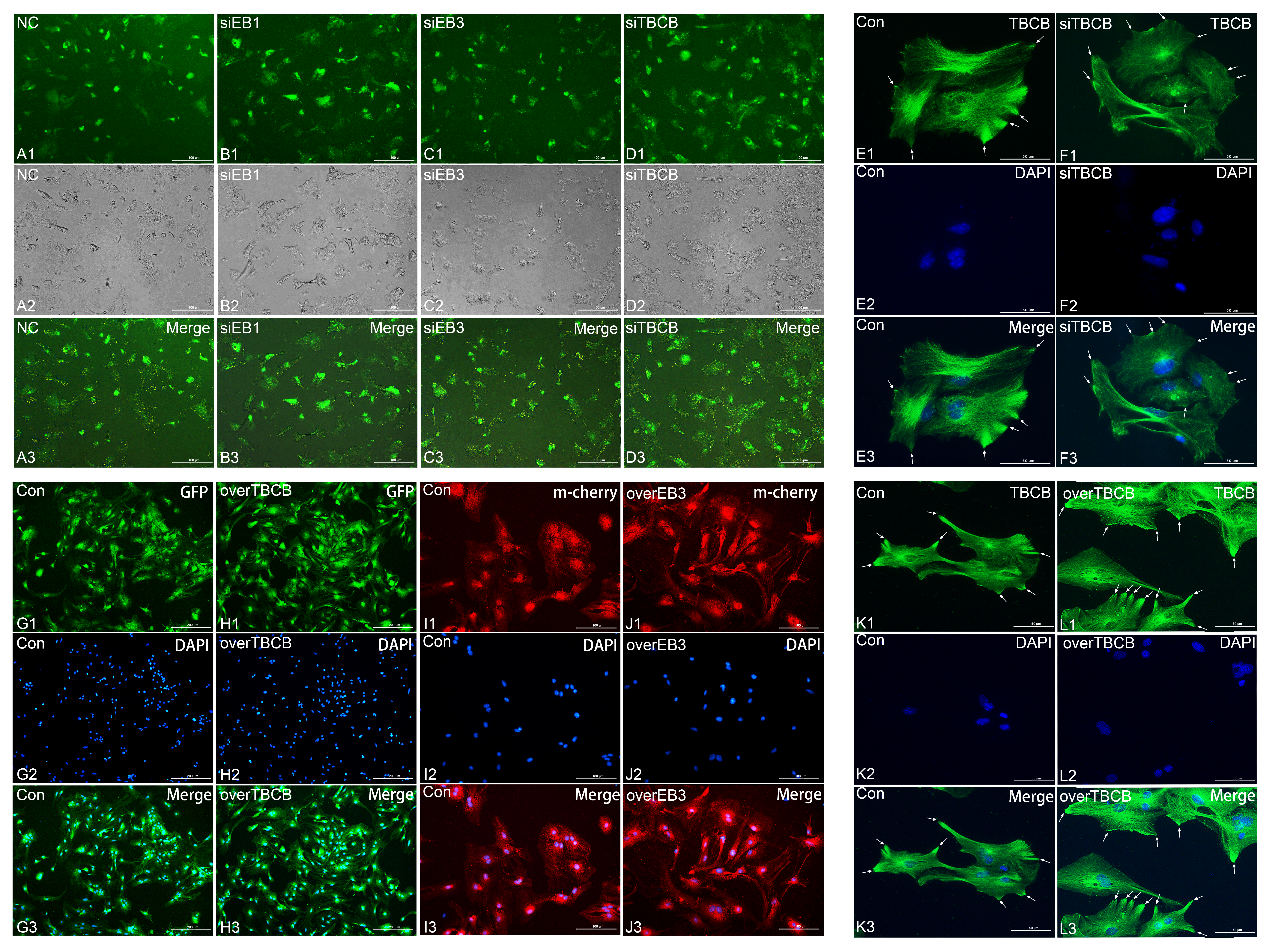


**Supplementary Figure 2.** Detection of the rate of siRNA transfection or lentivirus infection and Observation of morphological changes of astrocytes after TBCB was silenced with siRNA or was overexpressed with lentivirus at low magnification. **(A-D)** The transfection efficiency was more than 90% of siNC (A 1-3), siTBCB (B 1-3), siEB1 (C 1-3) and siEB3 (D 1-3). **(E, F)** Compared with the negative control group (E 1-3), after TBCB was scienced (F 1-3), the TBCB content decreased or disappeared, most astrocyte processes decreased significantly, or the formed processes collapsed or disappeared. **(G-J)** Anti-GFP/m-Cherry antibodies were used to detect the expression of TBCB because of the weak fluorescence of GFP/m-Cherry, a marker of viral infection. The infection efficiency was more than 95% of no-load (G, I 1-3), TBCB (H 1-3), or EB3 (J 1-3) overexpression lentivirus. **(K, L)** Compared with the no-load control group (K 1-3), after TBCB was overexpressed (L 1-3), The TBCB content increased.

## Supplementary Tables

**Supplementary Table 1.** Statistical tests used in the stud

| Corresponding figures | Prerequisites | Shapiro-Wilk test | Brown-Forsythe test or F test | Main test |
| --- | --- | --- | --- | --- |
| Fig. 1 E1 | Single factor, 3 groups, unpaired | Con: W(6)=0.9814  p=0.9582  30mM: W(6)=0.9081  p=0.4242  100mM: W(6)=0.9450  p= 0.6996 | Brown-Forsythe test ,  F (2,15)=0.3149  P=0.7346 | ANOVA, Tukey test,  Con vs. 30mM, Mean Diff. (15) = 2.817, p<0.0001  Con vs. 100mM, Mean Diff. (15) = 4.150, p<0.0001  30mM vs. 100mM, Mean Diff. (15) =1.333, p=0.0369 |
| Fig. 1 E2 | Single factor, 3 groups, unpaired | Con: W(6)=0.9257  p=0.5474  30mM: W(6)=0.9122  p=0.4513  100mM: W(6)=0.9228  p= 0.5259 | Brown-Forsythe test ,  F (2,15)=0.3912  P=0.6830 | ANOVA, Tukey test,  Con vs. 30mM, Mean Diff. (15) = 30.30, p<0.0001  Con vs. 100mM, Mean Diff. (15) = 47.13, p<0.0001  30mM vs. 100mM, Mean Diff. (15) =16.83, p=0.0110 |
| Fig. 1 E3 | Single factor, 3 groups, unpaired | Con: W(6)=0.9340  p=0.6116  30mM: W(6)=0.9507  p=0.7457  100mM: W(6)=0.9566  p=0.7931 | Brown-Forsythe test ,  F (2,15)=2.747  P=0.0963 | ANOVA, Tukey test,  Con vs. 30mM, Mean Diff. (15) = 61.78, p<0.0001  Con vs. 100mM, Mean Diff. (15) = 80.25 p<0.0001  30mM vs. 100mM, Mean Diff. (15) =18.48, p=0.0273 |
| Fig. 1 E4 | Single factor, 3 groups, unpaired | Con: W(6)=0.9793  p=0.9478  30mM: W(6)=0.8452  p=0.1438  100mM: W(6)=0.9399  p=0.6587 | Brown-Forsythe test ,  F (2,15)=0.3014  P=0.7441 | ANOVA, Tukey test,  Con vs. 30mM, Mean Diff. (15) = 34.44, p=0.0002  Con vs. 100mM, Mean Diff. (15) = 51.50, p<0.0001  30mM vs. 100mM, Mean Diff. (15) =17.07, p=0.0370 |
| Fig. 1 E5 | Single factor, 3 groups, unpaired | Con: W(6)=0.9152  p=0.4716  30mM: W(6)=0.8838  p=0.2868  100mM: W(6)=0.8838  p= 0.2871 | Brown-Forsythe test ,  F (2,15)=0.4372  P=0.2871 | ANOVA, Tukey test,  Con vs. 30mM, Mean Diff. (15) = 26.10, p=0.0025  Con vs. 100mM, Mean Diff. (15) = 48.24, p<0.0001  30mM vs. 100mM, Mean Diff. (15) =22.14, p=0.0087 |
| Fig. 1 F2 | Single factor, 3 groups, unpaired | Con: W(7)=0.8135  p=0.0556  30mM: W(7)=0.8703  p=0.1868  100mM: W(7)= 0.9903  p= 0.9938 | Brown-Forsythe test ,  F (2,18)= 1.406  P= 0.2708 | ANOVA, Tukey test,  Con vs. 30mM, Mean Diff. (18) = 0.2615, p<0.0001  Con vs. 100mM, Mean Diff. (18) = 0.5103, p<0.0001  30mM vs. 100mM, Mean Diff. (18) = 0.2488, p<0.0001 |
| Fig. 1 F3 | Single factor, 3 groups, unpaired | Con: W(7)=0.8135  p=0.0556  30mM: W(7)=0.8703  p=0.1868  100mM: W(7)=0.9321  p=0.5691 | Brown-Forsythe test ,  F (2,18)= 2.298  P=0.1292 | ANOVA, Tukey test,  Con vs. 30mM, Mean Diff. (18) = 0.2615, p=0.0012  Con vs. 100mM, Mean Diff. (18) = 0.5531, p<0.0001  30mM vs. 100mM, Mean Diff. (18) = 0.2916, p=0.0004 |
| Fig. 1 F4 | Single factor, 3 groups, unpaired | Con: W(5)=0.9582  p=0.7956  30mM: W(5)=0.8376  p=0.1585  100mM: W(5)=0.8610  p=0.2319 | Brown-Forsythe test ,  F (2,12)=1.920  P= 0.1890 | ANOVA, Tukey test,  Con vs. 30mM, Mean Diff. (12) = 0.3293, p=0.0054  Con vs. 100mM, Mean Diff. (12) = 0.6348, p<0.0001  30mM vs. 100mM, Mean Diff. (12) =0.3055, p=0.0089 |
| Fig. 1 G1 | Single factor, 3 groups, unpaired | Con: W(9)=0.9071  p=0.2960  30mM: W(9)=0.8957  p=0.2282  100mM: W(9)=0.9785  p=0.9559 | Brown-Forsythe test ,  F (2,24)=0.6887  P= 0.5119 | ANOVA, Tukey test,  Con vs. 30mM, Mean Diff. (24) = 0.001670, p=0.0127  Con vs. 100mM, Mean Diff. (24) = 0.003037, p<0.0001  30mM vs. 100mM, Mean Diff. (24) =0.001368, p=0.0449 |
| Fig. 1 G2 | Single factor, 3 groups, unpaired | Con: W(3)=0.9071  p=0.2960  30mM: W(3)=0.8623  p=0.2740  100mM: W(3)=0.9998  p=0.9743 | Brown-Forsythe test ,  F (2,12)=0.4282  P= 0.6701 | ANOVA, Tukey test,  Con vs. 30mM, Mean Diff. (6) = 0.0003306, p=0.0004  Con vs. 100mM, Mean Diff. (6) = 0.0005023, p<0.0001  30mM vs. 100mM, Mean Diff. (6) =0.0001717, p=0.0114 |
|  |  |  |  |  |
| Fig. 2 E1 | Single factor, 2 groups, unpaired | Con: W(6)=0.8965  p=0.3537  siTBCB: W(6)=0.8757  p=0.2497 | F test,  F (5,5)=1.159  P=0.8757 | Unpaired t test, t(10)=4.764,p=0.0008 |
| Fig. 2 E2 | Single factor, 2 groups, unpaired | Con: W(6)=0.8953  p=0.3470  siTBCB: W(6)=0.9355  p=0.6235 | F test,  F (5,5)=1.124  P=0.9012 | Unpaired t test, t(10)=3.819,p=0.0034 |
| Fig. 2 E3 | Single factor, 2 groups, unpaired | Con: W(6)=0.9520  p=0.7565  siTBCB: W(6)=0.9559  p=0.7880 | F test,  F (5,5)=1.000  P=0.9997 | Unpaired t test, t(10)=5.767,p=0.0002 |
| Fig. 2 E4 | Single factor, 2 groups, unpaired | Con: W(6)=0.9376  p=0.6400  siTBCB: W(6)=0.8887  p=0.3114 | F test,  F (5,5)=1.169  P=0.8682 | Unpaired t test, t(10)=4.657,p=0.0009 |
| Fig. 2 E5 | Single factor, 2 groups, unpaired | Con: W(6)=0.9570  p=0.7963  siTBCB: W(6)=0.9295  p=0.5765 | F test,  F (5,5)=1.746  P=0.5558 | Unpaired t test, t(10)=4.853,p=0.0007 |
| Fig. 2 F2 | Single factor, 2 groups, unpaired | TBCB:  Con: W(6)= 0.9345  p= 0.6151  siTBCB: W(6)= 0.9196  p= 0.5025  α-tubulin:  Con: W(6)= 0.9006  p= 0.3776  siTBCB: W(6)= 0.9310  p= 0.5878 | F test,  TBCB:  F (5,5)=5.168  P= 0.0957  α-tubulin:  F (5,5)=2.879  P= 0.2706 | Unpaired t test,  TBCB:  t(10)=7.191,p <0.0001  α-tubulin:  t(10)=6.091,p= 0.0001 |
| Fig. 2 G | Single factor, 2 groups, unpaired | Con: W(6)=0.9289  p=0.5713  siTBCB: W(6)=0.8725, p=0.2363 | F test,  F (5,5)=6.384  P=0.0630 | Unpaired t test, t(10)=6.033,p=0.0001 |
| Fig. 2 H1 | Single factor, 2 groups, unpaired | Con: W(6)=0.8497  p=0.1564  overTBCB: W(6)=0.9261  p=0.5504 | F test,  F (5,5)=2.023  P=0.4579 | Unpaired t test, t(10)=3.023,p=0.0128 |
| Fig. 2 H2 | Single factor, 2 groups, unpaired | Con: W(6)=0.9607  p=0.8251  overTBCB: W(6)=0.9204  p=0.5079 | F test,  F (5,5)=5.509  P=0.0845 | Unpaired t test, t(10)=4.061,p=0.0023 |
| Fig. 2 H3 | Single factor, 2 groups, unpaired | Con: W(6)=0.9302  p=0.5818  overTBCB: W(6)=0.9829  p=0.9651 | F test,  F (5,5)=3.136  P=0.2353 | Unpaired t test, t(10)=3.840,p=0.0033 |
| Fig. 2 H4 | Single factor, 2 groups, unpaired | Con: W(6)=0.9866  p=0.9792  overTBCB: W(6)=0.9804  p=0.9534 | F test,  F (5,5)=1.399  P=0.7213 | Unpaired t test, t(10)=3.356,p=0.0073 |
| Fig. 2 H5 | Single factor, 2 groups, unpaired | Con: W(6)=0.9707  p=0.8970  overTBCB: W(6)=0.9235  p=0.5312 | F test,  F (5,5)=1.077  P= 0.9372 | Unpaired t test,  t(10)=2.265,p=0.0469 |
| Fig. 2 I2 | Single factor, 2 groups, unpaired | TBCB:  Con: W(7)= 0.9680  p= 0.8835  overTBCB: W(7)= 0.9036  p= 0.3534  α-tubulin:  Con: W(6)= 0.9290  p= 0.5727  overTBCB: W(6)= 0.8811  p= 0.2740 | F test,  TBCB:  F (6,6)=4.302  P= 0.0991  α-tubulin:  F (5,5)=3.981  P= 0.1557 | Unpaired t test,  TBCB:  t(12)=5.707,p <0.0001  α-tubulin:  t(10)=5.079,p= 0.0005 |
| Fig. 2 J | Single factor, 2 groups, unpaired | Con: W(5)=0.8800  p=0.3092  overTBCB: W(5)=0.9314  p=0.6057 | F test,  F (5,5)=2.778  P= 0.3462 | Unpaired t test,  t(10)=5.748,p=0.0004 |
|  |  |  |  |  |
| Fig. 3 G1 | Single factor, 2 groups, unpaired | Con: W(6)=0.9463  p=0.7105  siEB1: W(6)=0.9330  p=0.6031 | F test,  F (5,5)=1.867  P=0.5098 | Unpaired t test,  t(10)=5.621,p=0.0002 |
| Fig. 3 G2 | Single factor, 2 groups, unpaired | Con: W(6)=0.8897  p=0.3166  siEB1: W(6)=0.9020  p=0.3860 | F test,  F (5,5)=4.140  P=0.1451 | Unpaired t test, t(10)=6.162,p=0.0001 |
| Fig. 3 G3 | Single factor, 2 groups, unpaired | Con: W(6)=0.8562  p=0.1765  siEB1: W(6)=0.9065  p=0.4141 | F test,  F (5,5)=4.054  P=0.1507 | Unpaired t test, t(10)=5.669,p=0.0002 |
| Fig. 3 H2 | Single factor, 2 groups, unpaired | EB1:  Con: W(6)= 0.9848  p= 0.9728  siEB1: W(6)= 0.7946  p= 0.0525  α-tubulin:  Con: W(6)= 0.9814  p= 0.9585  siEB1: W(6)= 0.9648  p= 0.8557 | F test,  EB1:  F (5,5)=3.551  P= 0.1906  α-tubulin:  F (5,5)=1.386  P= 0.7289 | Unpaired t test,  EB1:  t(10)=9.963,p <0.0001  α-tubulin:  t(10)=6.118,p= 0.0001 |
| Fig. 3 I | Single factor, 2 groups, unpaired | Con: W(5)=0.9717  p=0.8859  siEB1: W(5)=0.9349  p=0.6300 | F test,  F (4,4)=2.144  P=0.4782 | Unpaired t test, t(8)=5.285,p=0.0007 |
| Fig. 3 J1 | Single factor, 2 groups, unpaired | Con: W(6)=0.9358  p=0.6254  siEB3: W(6)=0.8999  p=0.3731 | F test,  F (5,5)=3.074  P=0.7213 | Unpaired t test,  t(10)=5.824,p= 0.0002 |
| Fig. 3 J2 | Single factor, 2 groups, unpaired | Con: W(6)= 0.8744  p= 0.2441  siEB3: W(6)= 0.9608  p= 0.8256 | F test,  F (5,5)=2.045  P= 0.4511 | Unpaired t test,  t(10)=5.500,p= 0.0003 |
| Fig. 3 J3 | Single factor, 2 groups, unpaired | Con: W(6)= 0.9487  p= 0.7296  siEB3: W(6)= 0.9647  p= 0.8554 | F test,  F (6,6)=1.127  P= 0.8991 | Unpaired t test,  t(10)=5.144,p= 0.0004 |
| Fig. 3 K2 | Single factor, 2 groups, unpaired | EB3:  Con: W(8)= 0.8956  p= 0.2636  siEB3: W(8)= 0.8910  p= 0.2390  α-tubulin:  Con: W(6)= 0.9644  p= 0.8528  siEB3: W(6)= 0.9715  p= 0.9022 | F test,  EB3:  F (7,7)=1.430  P= 0.6485  α-tubulin:  F (5,5)=4.585  P=0.1201 | Unpaired t test,  EB3:  t(14)=22.22,p <0.0001  α-tubulin:  t(10)=5.490,p= 0.0003 |
| Fig. 3 L | Single factor, 2 groups, unpaired | Con: W(5)=0.9549  p=0.7719  siEB3: W(5)=0.9575  p=0.7904 | F test,  F (5,5)=3.390  P=0.2641 | Unpaired t test,  t(8)=6.325,p= 0.0002 |
| Fig. 3 M1 | Single factor, 2 groups, unpaired | Con: W(6)= 0.8673  p= 0.2155  overEB3: W(6)= 0.9807  p= 0.9551 | F test,  F (5,5)=2.825  P= 0.2789 | Unpaired t test,  t(10)=4.386,p=0.0014 |
| Fig. 3 M2 | Single factor, 2 groups, unpaired | Con: W(6)=0.8980  p=0.3621  overEB3: W(6)=0.9065  p=0.4138 | F test,  F (5,5)=6.486  P=0.0610 | Unpaired t test,  t(10)=4.474,p=0.0012 |
| Fig. 3 M3 | Single factor, 2 groups, unpaired | Con: W(6)=0.9212  p=0.5144  overEB3: W(6)=0.8971  p=0.3571 | F test,  F (6,6)=2.301  P=0.3816 | Unpaired t test,  t(10)=2.323,p=0.0426 |
| Fig. 3 N2 | Single factor, 2 groups, unpaired | EB3:  Con: W(7)= 0.9507  p= 0.7362  overEB3: W(7)= 0.8346  p=0.0884  α-tubulin:  Con: W(6)= 0.9593  p= 0.8140  overEB3: W(6)= 0.9845  p= 0.9718 | F test,  EB3:  F (7,7)=1.048  P= 0.9565  α-tubulin:  F (5,5)=1.350  P= 0.7499 | Unpaired t test,  EB3:  t(12)=6.875,p <0.0001  α-tubulin:  t(10)=4.225,p= 0.0018 |
| Fig. 3 O | Single factor, 2 groups, unpaired | Con: W(6)=0.8281  p=0.1347  overEB3: W(6)=0.9546  p=0.7701 | F test,  F (6,6)=1.196  P=0.8667 | Unpaired t test,  t(8)=5.223,p=0.0008 |
|  |  |  |  |  |
| Fig. 4 C1 | Single factor, 2 groups, unpaired | Con: W(6)=0.9463  p=0.7105  siEB1: W(6)=0.9330  p=0.6031 | F test,  F (5,5)=1.867  P=0.5098 | Unpaired t test,  t(10)=5.621,p=0.0002 |
| Fig. 4 C2 | Single factor, 2 groups, unpaired | Con: W(6)=0.8867  p=0.3011  siEB1: W(6)=0.9506  p=0.7455 | F test,  F (5,5)=5.021  P=0.1011 | Unpaired t test,  t(10)=5.105,p=0.0005 |
| Fig. 4 C3 | Single factor, 2 groups, unpaired | Con: W(6)=0.9081  p=0.4243  siEB1: W(6)=0.9107  p=0.4408 | F test,  F (5,5)=1.757  P=0.5512 | Unpaired t test,  t(10)=9.821,p<0.0001 |
| Fig. 4 D2 | Single factor, 2 groups, unpaired | EB1:  Con: W(6)= 0.9848  p= 0.9728  siEB1: W(6)= 0.7946  p= 0.0525  TBCB:  Con: W(6)=0.9648  p=0.8562  siEB1: W(6)= 0.8940  p=0.3395 | F test,  EB1:  F (5,5)=3.551  P= 0.1906  TBCB :  F (5,5)=1.020  P= 0.9833 | Unpaired t test,  EB1:  t(10)=9.963,p <0.0001  TBCB :  t(10)=5.105,p= 0.0005 |
| Fig. 4 E | Single factor, 2 groups, unpaired | EB1:  Con: W(5)=0.9717  p=0.8859  siEB1: W(5)=0.9349  p=0.6300 | F test,  EB1:  F (4,4)=2.144  P=0.4782 | Unpaired t test,  EB1:  t(8)=5.285,p=0.0007 |
|  |  | TBCB:  Con: W(5)= 0.9678  p= 0.8609  siEB1: W(5)= 0.9707  p= 0.8800 | TBCB:  F (4,4)=1.317  P=0.7963 | TBCB:  t(8)=5.392,p=0.0007 |
| Fig. 5 E1 | Single factor, 2 groups, unpaired | Con: W(6)=0.9358  p=0.6254  siEB3: W(6)=0.8999  p=0.3731 | F test,  F (5,5)=3.074  P=0.7213 | Unpaired t test,  t(10)=5.824,p= 0.0002 |
| Fig. 5 E2 | Single factor, 2 groups, unpaired | Con: W(6)=0.9711  p=0.9000  siEB3: W(6)=0.9341  p=0.6117 | F test,  F (5,5)=1.099  P=0.9202 | Unpaired t test,  t(10)=5.586,p=0.0002 |
| Fig. 5 E3 | Single factor, 2 groups, unpaired | Con: W(6)=0.8733  p=0.2396  siEB3: W(6)=0.9664  p=0.8675 | F test,  F (5,5)=2.334  P=0.3739 | Unpaired t test,  t(10)=5.701,p=0.0002 |
| Fig. 5 F2 | Single factor, 2 groups, unpaired | EB3:  Con: W(8)= 0.8956  p= 0.2636  siEB3: W(8)= 0.8910  p= 0.2390  TBCB:  Con: W(6)= 0.8604  p= 0.1904  siEB3: W(6)= 0.9379  p= 0.6420 | F test,  EB3:  F (7,7)=1.430  P= 0.6485  TBCB :  F (5,5)=1.699  P= 0.5751 | Unpaired t test,  EB3:  t(14)=22.22,p <0.0001  TBCB :  t(10)=6.856,p <0.0001 |
| Fig. 5 G | Single factor, 2 groups, unpaired | EB3:  Con: W(5)=0.9549  p=0.7719  siEB3: W(5)=0.9575  p=0.7904 | F test,  EB3:  F (5,5)=3.390  P=0.2641 | Unpaired t test,  EB3:  t(8)=6.325,p= 0.0002 |
|  |  | TBCB:  Con: W(5)= 0.9019  p= 0.4203  siEB3: W(5)= 0.9298  p= 0.5950 | TBCB:  F (4,4)=3.750  P=0.2286 | TBCB:  t(8)=5.287,p=0.0007 |
| Fig. 5 H1 | Single factor, 2 groups, unpaired | Con: W(6)= 0.8673  p= 0.2155  overEB3: W(6)= 0.9807  p= 0.9551 | F test,  F (5,5)=2.825  P= 0.2789 | Unpaired t test,  t(10)=4.386,p=0.0014 |
| Fig. 5 H2 | Single factor, 2 groups, unpaired | Con: W(6)= 0.9892  p= 0.9870  overEB3: W(6)= 0.9163  p= 0.4793 | F test,  F (5,5)=2.476  P=0.3423 | Unpaired t test,  t(10)=2.968,p= 0.0141 |
| Fig. 5 H3 | Single factor, 2 groups, unpaired | Con: W(6)=0.9312  p=0.5894  overEB3: W(6)=0.9693  p=0.8877 | F test,  F (5,5)=1.403  P=0.7190 | Unpaired t test,  t(10)=7.374,p<0.0001 |
| Fig. 5 I2 | Single factor, 2 groups, unpaired | EB3:  Con: W(7)= 0.9507  p= 0.7362  overEB3: W(7)= 0.8346  p=0.0884  TBCB:  Con: W(5)= 0.9875  p= 0.9701  overEB3: W(5)= 0.9044  p= 0.4347 | F test,  EB3:  F (7,7)=1.048  P= 0.9565  TBCB :  F (4,4)=1.488  P= 0.7095 | Unpaired t test,  EB3:  t(12)=6.875,p <0.0001  TBCB :  t(8)=6.268,p= 0.0002 |
| Fig. 5 J | Single factor, 2 groups, unpaired | EB3:  Con: W(5)=0.8281  p=0.1347  overEB3: W(5)=0.9546  p=0.7701 | F test,  EB3:  F (4,4)=1.196  P=0.8667 | Unpaired t test,  EB3:  t(8)=5.223,p=0.0008 |
|  |  | TBCB:  Con: W(5)= 0.9706  p= 0.8793  overEB3: W(5)= 0.9260  p= 0.5696 | TBCB:  F (4,4)=8.227  P=0.0654 | TBCB:  t(8)=6.143,p=0.0003 |
|  |  |  |  |  |
| Fig. 6 F1 | Single factor, 2 groups, unpaired | Con: W(6)=0.9463  p=0.7105  siEB1: W(6)=0.9330  p=0.6031 | F test,  F (5,5)=1.867  P=0.5098 | Unpaired t test,  t(10)=5.621,p=0.0002 |
| Fig. 6 F2 | Single factor, 2 groups, unpaired | Con: W(6)=0.9493  p=0.7346  siEB1: W(6)=0.9281  p=0.5653 | F test,  F (5,5)=3.282  P=0.2181 | Unpaired t test,  t(10)=8.290,p<0.0001 |
| Fig. 6 G2 | Single factor, 2 groups, unpaired | EB1:  Con: W(6)= 0.9848  p= 0.9728  siEB1: W(6)= 0.7946  p= 0.0525  EB3:  Con: W(6)= 0.8507  p= 0.1595  siEB1: W(6)= 0.8979  p=0.3619 | F test,  EB1:  F (5,5)=3.551  P= 0.1906  EB3:  F (5,5)=3.416  P= 0.2037 | Unpaired t test,  EB1:  t(10)=9.963,p <0.0001  EB3:  t(10)=4.667,p= 0.0009 |
| Fig. 6 H | Single factor, 2 groups, unpaired | EB1:  Con: W(5)=0.9717  p=0.8859  siEB1: W(5)=0.9349  p=0.6300 | F test,  EB1:  F (4,4)=2.144  P=0.4782 | Unpaired t test,  EB1:  t(8)=5.285,p=0.0007 |
|  |  | EB3:  Con: W(5)= 0.8742  p= 0.2839  siEB1: W(5)= 0.9746  p= 0.9040 | EB3:  F (4,4)=6.259  P=0.1034 | EB3:  t(8)=5.081,p=0.0010 |
| Fig. 6 I1 | Single factor, 2 groups, unpaired | Con: W(6)=0.9358  p=0.6254  siEB3: W(6)=0.8999  p=0.3731 | F test,  F (5,5)=3.074  P=0.7213 | Unpaired t test,  t(10)=5.824,p= 0.0002 |
| Fig. 6 I2 | Single factor, 2 groups, unpaired | Con: W(6)=0.9674  p=0.8745  siEB3: W(6)=0.9772  p=0.9370 | F test,  F (5,5)=1.117  P=0.9064 | Unpaired t test,  t(10)=6.354,p<0.0001 |
| Fig. 6 J2 | Single factor, 2 groups, unpaired | EB3:  Con: W(8)= 0.8956  p= 0.2636  siEB3: W(8)= 0.8910  p= 0.2390  EB1:  Con: W(6)= 0.9783  p= 0.9427  siEB3: W(6)= 0.8948  p= 0.3439 | F test,  EB3:  F (7,7)=1.430  P= 0.6485  EB1:  F (5,5)=1.145  P= 0.8857 | Unpaired t test,  EB3:  t(14)=22.22,p <0.0001  EB1:  t(10)=8.085,p <0.0001 |
| Fig. 6 K | Single factor, 2 groups, unpaired | EB3:  Con: W(5)=0.9549  p=0.7719  siEB3: W(5)=0.9575  p=0.7904 | F test,  EB3:  F (5,5)=3.390  P=0.2641 | Unpaired t test,  EB3:  t(8)=6.325,p= 0.0002 |
|  |  | EB1:  Con: W(5)= 0.8891  p= 0.3526  siEB3: W(5)= 0.8338  p= 0.1484 | EB1:  F (4,4)=1.036  P=0.9736 | EB1:  t(8)=7.099,p=0.0001 |
| Fig. 6 L1 | Single factor, 2 groups, unpaired | Con: W(6)= 0.8673  p= 0.2155  overEB3: W(6)= 0.9807  p= 0.9551 | F test,  F (5,5)=2.825  P= 0.2789 | Unpaired t test,  t(10)=4.386,p=0.0014 |
| Fig. 6 L2 | Single factor, 2 groups, unpaired | Con: W(6)=0.9011  p=0.3805  overEB3: W(6)= 0.9420  p= 0.6751 | F test,  F (5,5)=1.924  P=0.4900 | Unpaired t test,  t(10)=3.966,p= 0.0027 |
| Fig. 6 M2 | Single factor, 2 groups, unpaired | EB3:  Con: W(7)= 0.9507  p= 0.7362  overEB3: W(7)= 0.8346  p=0.0884  EB1:  Con: W(7)= 0.9088  p= 0.3876  overEB3: W(7)= 0.9123  p= 0.4121 | F test,  EB3:  F (7,7)=1.048  P= 0.9565  EB1:  F (4,4)=2.083  P= 0.3935 | Unpaired t test,  EB3:  t(12)=6.875,p <0.0001  EB1:  t(8)=8.579,p <0.0001 |
| Fig. 6 N | Single factor, 2 groups, unpaired | EB3:  Con: W(5)=0.8281  p=0.1347  overEB3: W(5)=0.9546  p=0.7701 | F test,  EB3:  F (4,4)=1.196  P=0.8667 | Unpaired t test,  EB3:  t(8)=5.223,p=0.0008 |
|  |  | EB1:  Con: W(5)= 0.9897  p= 0.9785  overEB3: W(5)= 0.9195  p= 0.5270 | EB1:  F (4,4)=1.328  P=0.7899 | EB1:  t(8)=4.536,p=0.0019 |
|  |  |  |  |  |
| Fig. 7 I1 | Single factor, 2 groups, unpaired | Con: W(6)=0.8953  p=0.3470  siTBCB: W(6)=0.9355  p=0.6235 | F test,  F (5,5)=1.124  P=0.9012 | Unpaired t test, t(10)=3.819,p=0.0034 |
| Fig. 7 I2 | Single factor, 2 groups, unpaired | Con: W(6)=0.9520  p=0.7565  siTBCB: W(6)=0.9559  p=0.7880 | F test,  F (5,5)=1.000  P=0.9997 | Unpaired t test, t(10)=5.767,p=0.0002 |
| Fig. 7 I3 | Single factor, 2 groups, unpaired | Con: W(6)=0.9108  p=0.4417  siTBCB: W(6)=0.9523  p=0.7589 | F test,  F (5,5)=1.507  P=0.6637 | Unpaired t test, t(10)=5.852,p=0.0002 |
| Fig. 7 I4 | Single factor, 2 groups, unpaired | Con: W(6)=0.9302  p=0.5818  overTBCB: W(6)=0.9829  p=0.9651 | F test,  F (5,5)=3.136  P=0.2353 | Unpaired t test, t(10)=3.840,p=0.0003 |
| Fig. 7 J2 | Single factor, 2 groups, unpaired | TBCB:  Con: W(6)= 0.9345  p= 0.6151  siTBCB: W(6)= 0.9196  p= 0.5025  EB1:  Con: W(6)= 0.9450  p= 0.6999  siTBCB: W(6)= 0.8793  p= 0.2660  EB3:  Con: W(6)= 0.9166  p= 0.4812  siTBCB: W(6)= 0.8683  p= 0.2194 | F test,  TBCB:  F (5,5)=5.168  P= 0.0957  EB1:  F (5,5)=1.809  P= 0.5312  EB3:  F (5,5)=1.592  P= 0.6224 | Unpaired t test,  TBCB:  t(10)=7.191,p <0.0001  EB1:  t(10)=9.754,p <0.0001  EB3:  t(10)=2.726,p= 0.0213 |
| Fig. 7 K | Single factor, 2 groups, unpaired | TBCB:  Con: W(6)=0.9289  p=0.5713  siTBCB: W(6)=0.8725, p=0.2363  EB1:  Con: W(6)= 0.8896  p= 0.3159  siTBCB: W(6)= 0.9318  p= 0.5944  EB3:  Con: W(5)= 0.9046  p= 0.4017  siTBCB: W(5)= 0.9378  p= 0.6414 | F test,  TBCB:  F (5,5)=6.384  P=0.0630  EB1:  F (5,5)=2.916  P= 0.2651  EB3:  F (4,4)=1.603  P= 0.6170 | Unpaired t test,  TBCB:  t(10)=6.033,p=0.0001  EB1:  t(10)=5.247,p =0.0004  EB3:  t(8)=3.074,p= 0.0118 |
|  |  |  |  |  |
| Fig. 7 L1 | Single factor, 2 groups, unpaired | Con: W(6)=0.9607  p=0.8251  overTBCB: W(6)=0.9204  p=0.5079 | F test,  F (5,5)=5.509  P=0.0845 | Unpaired t test, t(10)=4.061,p=0.0023 |
| Fig. 7 L2 | Single factor, 2 groups, unpaired | Con: W(6)=0.9302  p=0.5818  overTBCB: W(6)=0.9829  p=0.9651 | F test,  F (5,5)=3.136  P=0.2353 | Unpaired t test, t(10)=3.840,p=0.0033 |
| Fig. 7 L3 | Single factor, 2 groups, unpaired | Con: W(6)=0.9234  p=0.5298  overTBCB: W(6)=0.9401  p=0.6598 | F test,  F (5,5)=6.882  P=0.0540 | Unpaired t test, t(10)=3.623,p=0.0047 |
| Fig. 7 L4 | Single factor, 2 groups, unpaired | Con: W(6)=0.8866  p=0.3010  overTBCB: W(6)=0.8984  p=0.3648 | F test,  F (5,5)= 2.959  P=0.2590 | Unpaired t test, t(10)=3.793,p=0.0035 |
| Fig. 7 M2 | Single factor, 2 groups, unpaired | TBCB:  Con: W(7)= 0.9680  p= 0.8835  overTBCB: W(7)= 0.9036  p= 0.3534  EB1:  Con: W(6)= 0.9868  p= 0.9800  siTBCB: W(6)= 0.9520  p= 0.1567  EB3:  Con: W(6)= 0.9851  p= 0.9741  siTBCB: W(6)= 0.8677  p= 0.2171 | F test,  TBCB:  F (6,6)=4.302  P= 0.0991  EB1:  F (5,5)=2.408  P= 0.3568  EB3:  F (5,5)=1.612  P= 0.6129 | Unpaired t test,  TBCB:  t(12)=5.707,p <0.0001  EB1:  t(10)=6.091,p= 0.0001  EB3:  t(10)=6.100,p= 0.0001 |
| Fig. 7 N | Single factor, 2 groups, unpaired | Con: W(5)=0.8800  p=0.3092  overTBCB: W(5)=0.9314  p=0.6057 | F test,  F (5,5)=2.778  P= 0.3462 | Unpaired t test,  t(10)=5.748,p=0.0004 |
|  |  |  |  |  |
| Fig. 8 D1 | Single factor, 3 groups, unpaired | Con: W(6)=0.9165  p=0.4807  TPA: W(6)=0.8851  p=0.2934  U0126: W(6)=0.8934  p=0.3367 | Brown-Forsythe test ,  F (2,15)=0.9166  P=0.4211 | ANOVA, Tukey test,  Con vs. TPA, Mean Diff. (12) = -1.600 , p=0.0235  Con vs. U0126, Mean Diff. (12) =4.067 , p<0.0001  TPA vs. U0126, Mean Diff. (12) =5.667, p<0.0001 |
| Fig. 8 D2 | Single factor, 3 groups, unpaired | Con: W(6)=0.8512  p=0.1609  TPA: W(6)=0.9059  p=0.4100  U0126: W(6)=0.9282  p= 0.5662 | Brown-Forsythe test ,  F (2,15)=0.9683  P=0.4022 | ANOVA, Tukey test,  Con vs. TPA, Mean Diff. (12) = -17.13, p=0.0325  Con vs. U0126, Mean Diff. (12) = 50.98, p<0.0001  TPA vs. U0126, Mean Diff. (12) = 68.11, p<0.0001 |
| Fig. 8 D3 | Single factor, 3 groups, unpaired | Con: W(6)=0.9728  p=0.9108  TPA: W(6)=0.8684  p=0.2199  U0126: W(6)=0.9590  p= 0.8121 | Brown-Forsythe test ,  F (2,15)=1.648  P=0.2254 | ANOVA, Tukey test,  Con vs. TPA, Mean Diff. (12) = -17.75, p=0.0152  Con vs. U0126, Mean Diff. (12) = 0.3897, p<0.0001 |
| Fig. 8 D4 | Single factor, 3 groups, unpaired | Con: W(6)=0.9218  p=0.5184  TPA: W(6)=0.8599  p=0.1889  U0126: W(6)=0.9141  p= 0.4639 | Brown-Forsythe test ,  F (2,15)=0.9751  P=0.3998 | ANOVA, Tukey test,  Con vs. TPA, Mean Diff. (12) =  -19.52 p=0.0002  Con vs. U0126, Mean Diff. (12) = 10.49, p=0.0260 |
| Fig. 8 D5 | Single factor, 3 groups, unpaired | Con: W(6)=0.7985  p=0.0570  TPA: W(6)=0.8432  p=0.1385  U0126: W(6)=0.9686  p= 0.8829 | Brown-Forsythe test ,  F (2,12)=0.6047  P=0.5621 | ANOVA, Tukey test,  Con vs. TPA, Mean Diff. (12) = -12.54, p=0.0255  Con vs. U0126, Mean Diff. (12) = 14.69, p=0093 |
| Fig. 8 E2 | Single factor, 3 groups, unpaired | Con: W(5)=0.9530  p=0.7584  TPA: W(5)=0.8439  p=0.1759  U0126: W(5)=0.9374  p= 0.6478 | Brown-Forsythe test ,  F (2,12)=0.6047  P=0.5621 | ANOVA, Tukey test,  Con vs. TPA, Mean Diff. (12) = 0.2065 , p=0.0076  Con vs. U0126, Mean Diff. (12) = 0.3897 , p<0.0001  TPA vs. U0126, Mean Diff. (12) = 0.1832 , p=0.0162 |
| Fig. 8 E3 | Single factor, 3 groups, unpaired | Con: W(4)=0.7803  p=0.0714  TPA: W(4)=0.7784  p=0.0688  U0126: W(4)=0.9849  p= 0.9300 | Brown-Fo rsythe test ,  F (2,9)=0.2990  P=0.7486 | ANOVA, Tukey test,  Con vs. TPA, Mean Diff. (9) = 0.1535, p=0.2764  Con vs. U0126, Mean Diff. (9) = 0.07255 , p=0.7247  TPA vs. U0126, Mean Diff. (9) = -0.08093, p=0.6722 |
| Fig. 8 E4 | Single factor, 3 groups, unpaired | Con: W(6)=0.8540  p=0.1695  TPA: W(6)=0.9251  p=0.5425  U0126: W(6)=0.9448  p= 0.6982 | Brown-Forsythe test ,  F (2,15)=1.454  P=0.2648 | ANOVA, Tukey test,  Con vs. TPA, Mean Diff. (15) = 0.07759 , p=0.6883  Con vs. U0126, Mean Diff. (15) = 0.1314 , p=0.3595  TPA vs. U0126, Mean Diff. (15) = 0.05384 , p=0.8333 |
| Fig. 8 F2 | Single factor, 2 groups, unpaired | Con: W(6)=0.8432  p=0.1385  siTBCB: W(6)=0.9355  p=0.6235 | F test,  F (5,5)=1.241  P=0.8183 | Unpaired t test, t(10)=5.091,p=0.0005 |
| Fig. 8 F3 | Single factor, 2 groups, unpaired | Con: W(3)=0.8829  p=0.3329  siTBCB : W(3)=0.9100  p=0.4181 | F test,  F (2,2)=1.134  P=0.9373 | Unpaired t test, t(4)=1.452,p=0.2200 |
| Fig. 8 F4 | Single factor, 2 groups, unpaired | Con: W(4)=0.8928  p=0.3964  siTBCB : W(4)=0.7998  p=0.1019 | F test,  F (3,3)=1.756  P=0.6552 | Unpaired t test, t(6)=0.6425,p=0.5443 |
| Fig. 8 G2 | Single factor, 2 groups, unpaired | Con: W(6)=0.9066  p=0.4145  overTBCB : W(6)=0.9500  p=0.7399 | F test,  F (5,5)=5.317  P=0.0905 | Unpaired t test, t(10)=5.389,p=0.0003 |
| Fig. 8 G3 | Single factor, 2 groups, unpaired | Con: W(5)=0.8880  p=0.3474  overTBCB: W(5)=0.8871  p=0.3429 | F test,  F (4,4)=2.818  P=0.3398 | Unpaired t test, t(8)=5.800,p=0.5779 |
| Fig. 8 G4 | Single factor, 2 groups, unpaired | Con: W(5)=0.9450  p=0.7016  overTBCB: W(5)=0.9199  p=0.5291 | F test,  F (4,4)=1.461  P=0.7223 | Unpaired t test, t(8)=0.2342,p=0.8207 |
| Fig. 8 H2 | Single factor, 3 groups, unpaired | Con: W(8)=0.9437  p=0.6473  TPA: W(8)=0.9134  p=0.3787  U0126: W(8)=0.8893  p= 0.2304 | Brown-Forsythe test ,  F (2,21)=1.426  P=0.2627 | ANOVA, Tukey test,  Con vs. TPA, Mean Diff. (21) =-0.2522, p=0.0079  Con vs. U0126, Mean Diff. (21) =0.3920, p<0.0001 |
| Fig. 8 H3 | Single factor, 3 groups, unpaired | Con: W(9)=4.162  p=0.1248  TPA: W(9)=0.3445  p=0.8418  U0126: W(9)=3.225  p= 0.1994 | Brown-Forsythe test ,  F (2,24)=3.019  P=0.0677 | ANOVA, Tukey test,  Con vs. TPA, Mean Diff. (24) =-0.3104, p=0.0003  Con vs. U0126, Mean Diff. (24) =0.2474, p=0.0033 |
| Fig. 8 H4 | Single factor, 3 groups, unpaired | Con: W(4)=0.9726  p=0.8575  TPA: W(4)=0.9774  p=0.8863  U0126: W(4)=0.9683  p= 0.8307 | Brown-Forsythe test ,  F (2,9)=0.6358  P=0.5517 | ANOVA, Tukey test,  Con vs. TPA, Mean Diff. (15) = -0.4402, p=0.0010  Con vs. U0126, Mean Diff. (15) = 0.3927, p=0.0022 |
